# Optimizing for happiness in EU technology policy to repay public subsidies for AI

**DOI:** 10.1098/rsos.261323

**Published:** 2026-10-21

**Authors:** Sandra Wachter, Brent Mittelstadt, Daria Onitiu, Netta Weinstein

**Affiliations:** ^1^ Hasso-Plattner-Institute, University of Potsdam, Potsdam, Germany; ^2^ Oxford Internet Institute, University of Oxford, Oxford, UK; ^3^ Weizenbaum-Institut eV, Berlin, Germany; ^4^ School of Psychology and Clinical Language Sciences, University of Reading, Reading, UK

**Keywords:** artificial intelligence, technology policy, technology law, European Union, happiness, well-being, law, policy, subsidies, regulation

## Abstract

Backlash against the artificial intelligence (AI) industry is growing globally in recognition of the high costs and highly uncertain risks borne by society. General-purpose AI systems are sold predominantly on the basis of economic benefits, with little attention given to social or non-financial benefits. Policymakers tasked with regulating AI while balancing European Union (EU) rights and values with innovation and free market competition face a seemingly impossible task. A change of priorities is urgently needed in EU tech policy. Current policy predominantly focuses on risk mitigation rather than delivering socially beneficial technologies. EU tech policy should be rebalanced in favour of societal interests owing to the substantial (in)direct subsidies paid to support the AI industry via tax breaks, public funding, privileged access to public lands, resources, data and infrastructure and widespread deregulation. We propose adding a third goal to rebalance EU AI policy-making: happiness. When policymakers are deciding whether subsidies should be granted for the AI industry, they should require companies to concretely predict measurable benefits for happiness, and hold them accountable for delivering on their predictions. To unpack this proposal, we examine decades of research in psychology to identify key determinants of happiness, focusing on three widely accepted traits: relatedness, competence and autonomy.

## 1. Introduction

The tale of Perseus and Medusa is a classic of Greek mythology. Perseus, son of divine parentage, is a strong and fearless hero who journeys to slay Medusa, a feared monster whose mere gaze can turn living beings into stone.

Yet on closer inspection, the tale of a seemingly self-made hero ridding the world of evil is much more complicated [1]. Perseus did not work alone—he had the benefit of divine intervention throughout his journey. He received a cap of invisibility from Hades, winged shoes from Hermes, a sword from Hephaestus and a shield, enchanted bag and strategic counsel from Athena. Perseus could not have slain Medusa by himself, and yet he is traditionally hailed as a self-made hero, an aspirational symbol of Greek heroism [1].

Today, similar myths are crafted about the founders and chief executive officers of major technology companies. These self-invented quasi-gods are said to make the impossible possible. They wield the power to shape society through their products and innovation, anticipating and solving problems the public cannot yet see.

However, just like Perseus, these ‘self-made’ billionaires rely on substantial outside help. Beyond their investors, mentors, employees and others, tech leaders rely on substantial ‘divine’ assistance from the state and public. More often than not, this help is given without consulting the public who ultimately pays for it. Whereas Perseus would not dream of ignoring his divine patrons, tech leaders readily pretend their public benefactors do not exist, perhaps to protect the myth of the ‘self-made’ genius.

This article aims to challenge this myth and start to answer three critical questions:

— How do European Union (EU) citizens and residents subsidize the age of artificial intelligence (AI)?
— What are they promised in return?
— How can tech policy ensure the public receives a fair share of benefits?

Our focus is on AI-relevant tech policy and the AI industry in the EU.^1^ As a region known (rightly or wrongly) for having very stringent technology regulation, it would be reasonable to expect it to have the most favourable balance between industrial and societal interests.

The public is in all but name a shareholder of AI companies. We first map some of the subsidies handed out on behalf of the public to make companies successful in the EU, including tax breaks, governmental funding, tariffs on foreign goods, access to public infrastructure, selling or lending land to build data centres and deregulation of labour, data protection, environmental and intellectual property (IP) laws. In particular, the recently proposed Digital Omnibus grants the AI industry substantial regulatory relief justified by vague promises of economic growth and increased competitiveness. Next, we review the predicted benefits of AI according to AI companies: economic growth, support for innovation^2^ and increased productivity and competitiveness of businesses and markets. In contrast, we find that the predicted social benefits are highly uncertain, resist precise measurement (and therefore future enforcement or redress) and are of dubious value.

We then review current tech policy to understand how predicted benefits and risks are balanced to protect EU values and rights (i.e. societal interests) while still enabling innovation (i.e. industrial interests). We find that EU tech policy generally takes a risk mitigation approach, which aims to measure and minimize the risks of new technologies, and thereby strike a balance between individual and social interests on the one hand, and industrial interests on the other. Policymakers assume that innovation will be socially beneficial and therefore justify its risks, but fail to ensure this balance in practice or demand concrete benefits from the AI industry. This is problematic as EU policymakers have a mandate to act in the best interests of the public, but are currently doing so under extreme uncertainty.^3^

We argue that this strong risk mitigation approach is ill-suited to address the predicted risks of AI in a holistic manner. The key problem is that less risky technologies are not necessarily socially beneficial, so continuing mainly with this current risk mitigation and balancing approach will not guarantee AI will be socially beneficial. This outcome is unacceptable given the actual, substantial costs already imposed on the public via subsidies in the name of innovation.

Current policy targets specific, concrete, measurable risks and how best to manage and mitigate them, but struggles to counteract less tangible, well-defined or acute risks. Many of the benefits and risks posed by AI are vague, open-ended, cumulative, longitudinal and difficult to measure. To continue serving its intended function, EU tech policy must adapt to address them. This is not to say that the current risk-based approach needs to be abandoned, but rather that it must be complemented with aspirational and positive goals that work to counteract the vaguely defined, open-ended risks posed by general-purpose AI.

To ensure EU tech policy continues to function as intended, we propose a third goal to guide current and future policy: happiness.

Current policy is defined by two goals: (i) fundamental and individual rights and EU values, and (ii) innovation and free market competition. Risks posed to the first by the second must be balanced through rules and restrictions on technology development, deployment and governance. The weakness of this approach is that risks must be well-defined and measurable to be mitigated through regulatory measures. We advocate for a third, aspirational goal to be added: happiness. This additional goal will serve as a precautionary safeguard to balance against the highly uncertain and under-researched cumulative effects of AI on society. We focus on happiness as a subcategory of individual well-being. Happiness is an ideal policy goal that already features internationally in public policy-making (appendix B), which reflects individual well-being and contributes to social well-being, as we discuss below.^4^

To unpack how and why this is a goal worth pursuing, we first examine a prominent meta-theory of human motivation and well-being within psychology—self-determination theory (SDT)—which has shown large global effects predicting happiness and other forms of well-being across 50 years of empirical research in psychology. ‘Relatedness’, ‘competence’ and ‘autonomy’ are three antecedents of happiness that have been described across diverse psychological theories in recent decades. They are viewed as innate psychological needs that serve as preconditions for people's happiness [2,3]. SDT provides a parsimonious explanatory framework to define how the environment supports or thwarts happiness, which extends to different domains of life (e.g. work and relationships) and levels of analysis (e.g. interpersonal, institutional and structural). These concepts can help determine whether AI can actually make people happier both in theory and practice, and thus whether it is actually worth the significant public and private investment seen in recent years from the perspective of individual happiness and social well-being.

We conclude by exploring how these psychological theories of happiness can be woven into policy considerations. We argue that happiness should be introduced as an additional public good that should be prioritized by legislators when regulating AI alongside traditional risk mitigation and economic considerations. Promoting and protecting the happiness of the public should be an overarching principle guiding the allocation of public resources (e.g. land, tax breaks, data, infrastructure and funding) as well as the drafting and enforcement of laws. Happiness should also guide efforts in AI literacy meant to enhance user autonomy, incentivize design choices when developing new products and feature on syllabi in schools.

## 2. Public subsidies for the AI industry

The AI industry enjoys a growing range of subsidies to improve its competitiveness and support deployment of AI products and services across the EU single market. We use the term ‘subsidy’ broadly to refer to actions and incentives by policymakers that benefit the AI industry while simultaneously weakening fundamental or individual rights protections or otherwise imposing tangible or opportunity costs on the public. Some of these actions are ‘direct’ subsidies involving governments giving money to companies, whereas others are ‘indirect’ subsidies or aid which bring financial or non-monetary benefits for companies. Subsidies take the form of economic, political and regulatory incentives granted on behalf of the general public. This advantageous environment has been constructed through a combination of lobbying, policy-making and (lack of) legal enforcement.

AI companies are subsidized by a range of mechanisms, including privileged access to public goods and resources such as access to governmental or public data [4] and buying or lending land to build data centres [5]; (free) access to public infrastructure, favourable research conditions and public investment in infrastructure (e.g. gigafactories), research and startups; favourable research conditions; various tax incentives [6, pp. 53–54], [7–11];^5^ tariffs on foreign goods;^6^ relaxation and deregulation of legal standards and duties in tech and human right laws [14], environmental law [15,16],^7^ copyright protection [18], labour law [19], [20, pp. 3–4] and merger and competition law [21], [22, pp. 6–9], [23–25]; and unregulated political capture [26,27]. All of these advantages enable their success in the market, and the public either directly or indirectly pays for them. In other words, these are the costs paid by the public to enable technological innovation.

Throughout this section, we focus on subsidies offered by the EU. We do, however, include some examples from other regions as they reveal the types of subsidies being granted to AI companies, as well as the way such subsidies can impact the public in the future.^8^

### 2.1. Simplified access to land, water, electricity and data

To further enable expansion of cloud computing and AI capacity, the EU has introduced plans to support the construction of data centres across Europe through various incentives and weakened regulatory requirements. Data centres create capacity for both private and public (e.g. scientific and governmental) computing and storage needs, and serve as essential infrastructure for many key Internet and computing services and platforms. However, where these incentives are intended to expand computing capacity specifically for generative AI products and services, questions must be asked about whether the technology's proposed benefits justify the costs imposed on the public (§3).

The EU's proposed Cloud and AI Development Act (CAIDA) aims to triple European data centre capacity over the next 5–7 years. CAIDA will fast-track building permits and ease access to energy infrastructure and financial aid for companies. The Act follows a December 2025 proposal to streamline the permitting process and exempt many tech infrastructure projects from environmental assessments entirely to meet ‘pressing geopolitical and competitiveness challenges’ [14, p. 1].^9^ These and related plans to weaken or not enforce existing environmental legal standards are predicted to cost the general public between €180 billion and €325 billion per year through forgone benefits, while saving affected companies €1 billion per year.^10^

Proposals to weaken environmental and permitting standards have faced significant criticism in recognition of their costs for the general public [5]. These types of incentives threaten to raise prices and limit general public access to scarce resources.^11^ Across Europe, residents already compete with data centre developers for water, land and electricity resources, for example, in Spain, Germany, the UK, Italy, The Netherlands, France as well as Ireland, where data centres already account for 22% of national annual electricity consumption and consume more than all urban households combined [5,32]. The general public is already losing access to (semi-)natural land (e.g. forests and agricultural land) and facing increased electricity and water demand from data centres that outstrips available supply [33]. In the USA, residents are losing access to clean water [34].

In many cases, governments justify these increased costs and competition with the employment opportunities and economic benefits data centres are predicted to bring to local and regional communities. Unfortunately, benefits often fail to materialize. In Romania and North Holland, jobs promised by data centre companies, which received financial and environmental incentives from local authorities, were extremely limited.^12^ In the USA, companies have received various tax exemptions worth tens of millions of dollars in the past to build data centres. These exemptions caused budgetary gaps for local governments and yet created only a handful of jobs in return,^13^ with some states currently reconsidering them [40].

New EU laws like the recast Energy Efficiency Directive (EED) will do little to correct the imbalanced incentives currently enjoyed by the AI industry. The EED creates environmental reporting duties but does not secure adequate public access, meaning companies can keep their sustainability figures secret [41–43]. It likewise fails to impose binding EU-wide efficiency targets for data centres, relying instead on Member States to set local targets and initial enforcement may be lax.^14^ The reporting metrics used are also deeply flawed and fail to capture the actual energy consumption and relative efficiency of data centres [46].

AI companies also benefit from widespread access to public datasets. Frontier and generative AI models in particular are trained on data scraped from the open Internet as well as public and private repositories of books, articles, academic papers and other written materials. Companies currently benefit from relaxed enforcement and interpretation of relevant copyright and IP laws [47–49], although court cases remain pending, concerning the rights of companies and copyright holders [50].

Access to public and government datasets is often provided through contracts to provide essential public IT services or infrastructure. DeepMind and Palantir, for example, have won controversial access to public health data in the UK to build platforms for the National Health Service [4,51,52]. In the EU, Google, Microsoft and Amazon Web Services are the main providers for cloud computing services for governments. Critics have warned that the current US administration will compel companies to transfer EU data to the USA, with such requests already rapidly increasing [53,54].^15^ Some Member States are already calling for divestment in the name of digital sovereignty [55].^16^ The public is rarely directly consulted or able to challenge such contracts even though it is their data being accessed and their rights being impacted.

### 2.2. Public funds and tax breaks

The EU is actively working to increase public spending on AI and data centres to improve competition and reduce reliance on US and Chinese companies. In 2025, the EU launched the InvestAI initiative to mobilize €200 billion of investment in AI infrastructure (e.g. gigafactories), research and startups, €50 billion of which comes directly from taxes [8,59,60]. A further €1 billion is being repurposed from existing financing programmes in support of the ‘Apply AI Strategy’, which aims to improve European productivity, competitiveness and technological sovereignty.^17^ This follows a 2025 policy proposal to enable the redirection of existing ‘unused’ tax funds to invest into ‘AI Gigafactory projects and facilities, and for the creation of multi-site gigafactories across multiple countries’ [62,63]. These decisions impose significant opportunity costs, as these are public funds that could be used elsewhere to create immediate, tangible benefits for the public.

Beyond direct financing, AI companies also benefit from significant tax breaks and exemptions, which lessen their tax burden and thus their contribution to the state and public goods. Profits from digital activities are often not taxed owing to many companies not having a physical presence in the EU [64]. AI companies nonetheless generate substantial revenue from the data of EU citizens and residents.

The need for a new tax system to close this gap has been previously recognized by the Organisation for Economic Co-operation and Development (OECD), which proposed a global minimum rate of effective corporate tax of 15% in 2021.^18^ This proposal was subsequently adopted by the EU and put into force in 2024 [65]. Many EU countries have also recently introduced separate digital services taxes.^19^ These taxes average between 3 and 5% and cover activities such as online advertising, data sales, digital intermediation services that facilitate the exchange of goods or services and on-demand audio-visual media services.^20^

While these appear to be promising steps taken by the EU to ensure multi-national AI companies pay their fair share towards public goods, the USA has successfully lobbied against them. As of 2026, US-based multi-national tech companies have been exempted from the OECD's 15% global corporate tax [66,67]. In July 2025, the EU abandoned plans to introduce an EU-wide digital tax, potentially owing to pressure from the Trump administration in trade deal negotiations happening in parallel [11]. US multi-national tech companies thus continue to enjoy a lower tax burden while expanding data centres and AI offerings across Europe.

### 2.3. Regulatory leniency

Regulatory frameworks are only as effective as the political willingness to enforce them. Tech companies have long complained that compliance with relevant frameworks can be complex, resource-intensive and prone to uncertainty owing to the vagueness of legal requirements. With newly introduced frameworks such as the Digital Services Act (DSA) and Digital Markets Act (DMA), many multi-national tech companies are going a step further in refusing or delaying compliance with many requirements. These companies are, thus far, only facing fines and investigations by the European Commission while still being allowed to operate in the EU single market despite planned or declared non-compliance.^21^ This leniency in enforcement can be considered yet another type of advantage enjoyed by major tech companies.

The DSA aims to reduce the social and systemic harms of online platforms and search engines, including misinformation, disinformation, negative impacts on political processes, child safety, gender-based violence, illegal content and human rights violations [69–72]. Early reports suggest many multi-national tech companies are refusing to fully comply with the DSA, with both the compliance picture and active investigations by the EU Commission changing rapidly [73–75].

With regard to disinformation and fact-checking obligations under the DSA's Code of Practice, for example, Microsoft and Google are reported to have refused to meet fact-checking obligations [73,76,77]. TikTok and Meta are currently under investigation by the European Commission [78,79], with the latter having ceased third-party fact-checking on Facebook, Instagram and Threads in the USA in early 2025 [80]. According to reporting from early 2025, Google, Microsoft and TikTok ‘have withdrawn from all commitments related to political advertising, citing their current ban on political ads’, and are likewise refusing to meet requirements to ‘develop, fund and collaborate with an independent third-party body to enable data access for researchers’ [73]. A September 2025 report from the European Fact-Checking Standards Network found that many platforms are either ignoring or formally abandoning fact-checking commitments but still presenting as signatories to the DSA Code of Practice [81].^22^

AI companies also enjoy leniency with regard to the DMA, which is intended to revive healthy market competition and reduce monopolistic behaviour. Many companies are considered ‘gatekeepers’ under the DMA, meaning they face a range of platform and service governance obligations. Initial analysis of compliance reports submitted by major tech companies, including Amazon, Microsoft and Meta, in 2026 found that they have thus far failed to make major changes to their compliance strategies as gatekeepers, and instead are taking a cautious and slow approach to compliance [82].

These are only a few recent examples of seemingly anti-competitive behaviour that went unpunished. AI companies routinely buy up their rivals before they become a threat (e.g. Meta buying WhatsApp and Instagram, and Google buying DoubleClick) [83]. While this behaviour is understandable from a business point of view, it is worrying from a consumer protection standpoint. Monopolies are a danger to ‘market competitiveness’ and ‘consumer welfare’ [84]. Competition authorities are tasked with investigating and preventing such mergers that would harm the market and consumers. Any failure to strongly enforce competition law can therefore be treated as a passive advantage enjoyed by tech companies at the cost of consumer welfare.

In Europe and the USA, authorities have generally treated potential tech mergers permissively in recent years. Examples include the EU Commission's approval of Google's acquisition of Photomath in 2023 [85] and Wiz in 2026 [21], Microsoft's acquisition of Activision Blizzard in 2023 [86] and Amazon's acquisition of MGM in 2022 [87]. While there are still cases where the Commission has prevented mergers such as the acquisition of iRobot by Amazon [88], many mergers proceed uninhibited [83,84,89]. The Microsoft–Mistral partnership also successfully avoided a merger investigation by the UK authorities in 2023 [90].

Emerging AI monopolies also appear to be receiving leniency in the USA. The AI industry is deeply circularly invested, with hundreds of billions of dollars being exchanged between 10 major AI companies [91,92]. For example, by 2023, both Amazon ($4 billion) and Google ($2 billion) invested heavily in Anthropic, a relatively new AI start-up [93]. As part of an anti-trust case against Google for potential monopolization of the search industry, in 2025 the US Department of Justice proposed that Google should be required to divest from AI competitors including Anthropic, but subsequently walked back the proposal [93].^23^ In 2026, Google announced a further $40 billion investment into Anthropic [95].

### 2.4. Deregulation of tech policy and political capture

Recent years have seen a strong push to weaken existing and proposed EU technology-relevant laws. The push for deregulation has come both internally from EU Member States such as Germany [96], France [97], Sweden [98] and Denmark [99], but also externally from the USA and multi-national technology companies [100]. Lobbying efforts have focused on revising and weakening the EU AI Act, DSA [101], privacy laws including the General Data Protection Regulation (GDPR) and proposed ePrivacy Regulation [102],^24^ non-discrimination law, liability frameworks [104,105] and environmental sustainability standards relevant to data centre construction [33].

A key result of these efforts is the newly proposed and heavily lobbied Digital Omnibus, which will replace or amend some of these and other laws with the stated goal of simplifying the legal landscape for technology companies to increase competitiveness, streamline legal compliance, support the growth of micro-, small- and medium-sized enterprises and thus unleash the ‘full potential’ of AI [106].^25^ If adopted, the Omnibus will create a much friendlier data protection environment for companies.^26^ It will establish (i) data protection privileges and exceptions for (commercial) research^27^ and the training of (general-purpose) AI models,^28^ (ii) relaxed data protection standards for pseudonymized data and biometric processing (i.e. special category data),^29^ (iii) tighter restrictions on access rights for data subjects,^30^ (iv) limitations on the definitional scope of personal data,^31^ and (v) weakened protections against automated decision-making.^32^

Other areas of EU law have also been weakened. A group of EU Member States blocked an amendment to non-discrimination law which would have strengthened protections against (AI-driven) discrimination based on religion, belief, disability, age and sexual orientation [110].^33^ The proposed AI Liability Directive, which would have complemented the existing Product Liability Directive with rules tailored to AI systems, suffered a similar fate [111], as did the ePrivacy Regulation, which was withdrawn following lobbying by advertising, telecoms and technology companies, as well as some EU Member States [103].

These trends are, of course, nothing new. Lobbying has strongly impacted the regulation of Internet platforms; a recent study by Falkner shows that, since 2022, the EU has consistently sided with the interests of the AI industry (i.e. platform providers) over the public [101]. A 2026 study by Birhane *et al.* [27] provides further evidence of political capture by the tech industry. While proponents of deregulation have hailed these trends as essential cutting of ‘red tape’ to facilitate innovation,^34^ human rights advocates have argued that they equally weaken individual rights by creating loopholes and gaps for industry to exploit [113].^35^

The ‘revolving door’ between industry and policy-making in recent decades has enabled the political capture essential to construct and maintain the subsidized environment for the tech industry discussed throughout this section. Recent notable examples in the EU include Member of the European Parliament (MEP) Aura Salla, the former Director of EU Policy at Meta from 2020 to 2023, who joined the European Parliament in 2024 and has been acting as rapporteur on the controversial Digital Omnibus proposal since 2026. Non-governmental organizations (NGOs) are calling for her removal from this position [26,115]. Further, Niamh Sweeney, who has held positions in tech companies such as Meta (2015–2021) and Stripe (2021–2022), was appointed in 2025 as Irish Data Protection Commissioner [116]. The UK also has many high-profile examples.^36^

## 3. What are the promised benefits of AI?

The AI industry benefits from many subsidies to enable innovation and deployment of AI products and services. At the same time, they argue that failing to deregulate will continue to stifle innovation and place the EU at a competitive disadvantage globally in the AI ‘arms race’. The common narrative that the ‘EU regulates while the world innovates’ is being deployed to support deregulation, premised on the unreliable claim that innovation and regulation cannot coexist [118–120]. The public thus faces high costs for enabling AI, but what is it promised in return?

To begin to understand the vision of AI currently being sold to (i) businesses, (ii) the public sector, and (iii) the general public, we reviewed a non-comprehensive sample of recent promotional materials (e.g. reports, sales materials, press releases, product briefings and other grey literature) published or sponsored by major AI companies including IBM, Google, DeepMind, Microsoft, OpenAI, Amazon Web Services, Nvidia and Anthropic (see appendix A for an overview of reviewed documents). Given their recency, these documents predominantly focus on general-purpose or generative AI. We find that the main selling points for AI across these companies and sectors are economic, while little attention is given to justifying the social and regulatory costs of the technology, and concrete details of socially beneficial use cases and their underlying mechanisms remain rare.

### 3.1. Promised benefits and use cases

The reviewed documents focused heavily on AI products and services for businesses. AI companies espouse broad visions for the future of AI driving substantial economic growth [121, p. 10], [122, pp. 4–5], enhanced productivity and competition [122, pp. 4–5], [123, p. 4], [124, p. 2], greater speed and efficiency by automating existing processes or work^37^ and improved data-driven insights and decision-making [121, p. 4]. Predicting the future economic impact of AI is of course difficult, but a 2023 study sponsored by Microsoft claimed that ‘[f]or every $1 companies invest in AI, they are realizing an average of $3.5 in return, and 5% of organizations worldwide are realizing an average of an average $8 in return’ [125, p. 2]. Similarly, OpenAI has claimed that ‘AI leaders have seen 1.5x faster revenue growth, 1.6x higher shareholder returns, and 1.4x better return on invested capital than their less advanced peers’ [126, p. 3].

Concrete and detailed use cases to substantiate these economic claims are rare in the reviewed documents. They instead focus on broad types of work (e.g. summarization, writing, data analysis, coding, visualizations and content creation) or business processes (e.g. personalized customer interactions, business operations, quality assurance and auditing) that can be improved [121, p. 4], [123, p. 4], [124, pp. 2, 6], [126, pp. 6–8, 11–24], [127, p. 1]. Benefits are predicted across sectors including medicine, telecommunications, retail, manufacturing, finance, product design and software development [122, p. 35], [127, p. 1], [128, p. 5].

AI companies are also interested in working with the public sector [129, p. 37]. Many already have significant partnerships with government departments to provide products, public services and research.^38^ Predictions made here echo those made for businesses, focusing on increasing productivity and efficiency of public bodies,^39^ reducing unemployment [132], streamlined and automated workflows [129, p. 37], [131], improved data-driven insights and analytics [129, p. 37] and improvements to infrastructure and citizen services.^40^ Work types to be automated include summarization [137, p. 11], question-answering and fact-finding (e.g. public-facing first response chatbots) [134], [137, p. 11], research [138, p. 4], [139], writing [138, p. 4] and mail sorting [134]. Again, detailed use cases are rare, with some companies highlighting high-level positive use cases in sectors such as health, science, education and government services [139, p. 3], [140].

The general public is less of a focal point in the reviewed documents. Again, companies make vague predictions about high-level benefits such as economic growth, employment opportunities, upskilling and work optimization [126, p. 3], [141]. The public is predicted to indirectly benefit from more efficient business and public processes, such as quicker answers to questions or access to information about government services [135,136].^41^ One of the most common predictions made by companies is that AI will improve customer satisfaction.^42^

It is worth remembering that the aforementioned predictions about the impact of AI on medicine, research, education, content creation and similar areas will have downstream impacts on the public, both positive and negative. Individuals may be empowered to do their jobs more quickly or efficiently but may also face greater cognitive fatigue or competition for employment [143,144]. Whether these effects are ultimately beneficial to the public remains to be seen.^43^

Across the reviewed documents, it is notable that the main selling points for (generative) AI are purely economic: economic growth, easier innovation and increased productivity and competitiveness. Companies offer limited discussion of non-economic benefits or the ‘public value’ offered by their technologies [145, p. 114].^44^ Socially beneficial use cases are rarely discussed, and typically at a high level with little detail or evidence of positive impacts for the general public.^45^ This gap is striking given the strong push to deregulate as well as the significant subsidies being given to the AI industry in the EU, all for the sake of enabling innovation. These choices come at the cost of public funds and fundamental rights, which is not reflected in the sales pitch of AI companies. Drawing inspiration from Prainsack's observation that the ‘public value’ of technologies is frequently left vague and contested [145, p. 114],^46^ we argue that public subsidies for AI require clear justification in these terms. Currently, it is not clear how the public will benefit from AI.

### 3.2. Will AI deliver economic growth?

The AI industry is clearly promising substantial economic growth. What remains unclear is how these predictions will fare in the future. Empirical evidence of the economic impact of AI remains nascent. It is nonetheless useful to review early signals to understand possible pathways forward for the technology.

Many analysts have already cast doubt on the scale of AI's potential economic contribution. A 2024 report by Goldman Sachs noted that returns on investment are thus far slow, and efficiency gains from AI do not outweigh the costs of training and deploying increasingly large models, which would need to solve much more complex tasks to be profitable [148]. A subsequent 2025 report speculated that AI may already be fuelling a financial bubble [149]. The gross domestic product (GDP) boost over the next 10 years in the USA is predicted to be modest [150]. Deloitte published a report in the same year stating that executives across the EU and Middle East do not believe that generative AI will deliver a satisfactory return on investment in the next 2–4 years [151]. Similarly, a 2025 report from the Massachusetts Institute of Technology (MIT) noted that ‘[d]espite $30–40 billion in enterprise investment into GenAI…95% of organizations are getting zero return’ and only ‘5% of integrated AI pilots are extracting millions in value’ [152, p. 3]. With regard to productivity, a possible predictor of future growth, a 2026 survey of 6000+ C-suite executives across the USA, UK, Germany and Australia revealed that 90% reported that AI had no impact on productivity or employment at their daily business [153].^47^ In contrast, some experiments have shown increases in productivity and satisfaction in writing and customer support tasks [155,156].

While all of the above is preliminary evidence, it is important to remember that industrial and societal economic growth are not the same. Many providers of AI products and services, and underlying hardware and infrastructure, are currently experiencing rapid increases in value and market share, which would suggest the technology is contributing to economic growth [157]. However, individual AI companies growing does not guarantee sustained economic growth across society. Economic growth is not the same as economic equality. In this respect, concerns must already be raised that AI's disruptive effects could widen economic inequalities [150].^48^

Companies are also taking steps to reduce their liability for (non-)financial harms caused by their AI products and services, which would appear to undermine their claims about the inevitability of financial and productivity benefits. Microsoft, for example, initially stated in their Terms of Service that Copilot should be used only for ‘entertainment purposes’, despite being heavily marketed at the time as a tool that should increase productivity.^49^ The terms and conditions further indemnify Microsoft against liability for inaccuracies or financial losses incurred from using Copilot. OpenAI, Google and Anthropic have added similar statements to their Terms of Service to shield themselves from potential liability.^50^

Of course, any analysis of general-purpose and generative AI's potential economic impact must necessarily be treated as incomplete and preliminary at this stage. With that said, preliminary analysis appears to cast doubt on these technologies delivering substantial economic growth in the near term. This uncertainty is troubling because, while economic growth (and equality) is not guaranteed, the social and financial costs paid by the public to subsidize the AI industry are already very real and significant.

### 3.3. The uncertain impact of AI on economic growth

Regardless of its likelihood, the potential economic growth brought about by AI remains a key selling point, enabling innovation, increased productivity and competitiveness and other social goods. These ends seem inherently valuable for society at first glance.^51^ However, the relationship between economic growth and social well-being is not simple. While growth can clearly contribute to public goods, for example, through greater taxation to fund improved public services (e.g. transportation and healthcare) or improved job opportunities for citizens,^52^ economists have also long studied the relationship and frequent tension between growth and well-being, for example, through the lens of ‘welfare economics’.^53^

Unlimited economic growth (through AI) is not necessarily desirable. Growth has instrumental value, measured by its contribution to goods such as liberty, security, basic needs (e.g. access to food, shelter, clothing) or time with friends, family and community [170, pp. 10–11], [173, pp. 832–833, 835]. Fulfilment of basic needs is a stronger predictor of well-being than income [174], while the contribution of wealth to happiness beyond certain thresholds varies by circumstance [175,176]. In other words, there may be such a thing as ‘enough’.^54^

Simultaneously, unfettered pursuit of growth in recent decades has led to worsening economic and social inequality across countries.^55^ This strongly indicates that economic growth from AI will not suddenly be equally shared across the people subsidizing it. Instead, it is likely that this pursuit will exacerbate both social and economic inequalities.^56^ For many economists and social theorists, many forms of inequality are inherently bad and should be eradicated through social policies and governmental action (for our proposal, see §7).^57^

The impact of economic growth brought about by AI on societal well-being is therefore an open question for the future despite confident but simplistic predictions from the AI industry. Economic growth can improve societal well-being if directed at the right societal goals by its creators, regulators and users.^58^ EU policymakers have a significant role to play here. Proactive decision-making can ensure the benefits of AI are more equally shared across society. The reactive approach currently taken towards AI policy-making unfortunately leaves much to be desired in this regard.

## 4. EU tech policy as risk mitigation

A current popular narrative, often advanced by industry and policymakers, claims that regulation stifles innovation, whereas deregulation, lax regulatory enforcement and subsidies enable it. Setting aside the fact that this narrative is contested [118,188], it is concerning that individuals and society bear the main costs and risks of ‘enabling innovation’ in these terms without any guaranteed benefits [189]. As a result, people are more exposed to the risks of AI and less able to seek legal recourse when harmed than would otherwise be the case. The willingness of governments and policymakers to grant subsidies on behalf of the public while being aware of these risks would suggest that they see technological innovation as inherently or instrumentally beneficial to society and worth the costs imposed. These beliefs are evident in the trade-offs between rights and innovation struck across EU tech policy, balancing enablers of innovation with protections for the vital interests of society and individual rights.

To map these trade-offs and assess the extent to which policymakers view innovation as inherently or instrumentally valuable, we review recent EU tech policy relevant to AI (table 1). These frameworks reveal how policymakers envision the role, value and risks of emerging technologies in society, including the risks of ‘hindering’ innovation through overly stringent regulation and failing to sufficiently invest public funds.^59^ The general strategy pursued across the reviewed frameworks is risk mitigation.^60^

**Table 1.** Reviewed EU technology regulatory frameworks.

EU regulatory framework	acronym	date (in force)	identifier
AI Act	AIA	2024	2024/1689
Copyright Directive	CD	2019	2019/790
Data Act	DA	2024	2023/2854
Data Governance Act	DGA	2022	2022/868
Digital Markets Act	DMA	2022	2020/842
Digital Omnibus	DO	2025*	2025/0360
Digital Services Act	DSA	2022	2022/2065
European Chips Act	ECA	2023	2023/1781
European Media Freedom Act	EMFA	2024	2024/1083
General Data Protection Regulation	GDPR	2018	2016/679
Markets in Crypto-Assets Regulation	MiCA	2024	2023/1114
Political Advertising Regulation	PAR	2025	2024/900
Product Liability Directive (updated)	PLD	2026	2024/2853

The reviewed frameworks predict two types of benefits: (i) general economic growth, competitive advantages and digital sovereignty, or (ii) sector-specific economic and productivity benefits. Concerning the former, examples include the GDPR enabling the free flow of data for economic growth [191, Recitals 2–3], the Copyright Directive preventing restrictions on technological development [192, Recital 3], MiCA focusing broadly on business and employment growth [193, Recitals 1–4], DSA harmonizing rules for responsible provision of online platforms and services across Member States to protect the internal market [194, Recitals 2, 4], as well as the AI Act [195, Recital 4], DMA [194, Recital 1], DO [106, Recitals 1–3], Data Act [196, Recitals 1–5] and Chips Act [197, Recitals 2–4] mentioning improved competitiveness and digital sovereignty. Perhaps unsurprisingly, these predictions tend to be vague or high-level. Specific mechanisms to drive economic growth are rarely discussed beyond affirmations of compliance with the EU's high regulatory standards as a sign of quality (and thus, competitiveness) for tech products and services [106, Recital 2].

Concerning the latter, the AI Act^61^ and DGA [198, Recitals 1–5] predict benefits across a wide range of publicly beneficial sectors including healthcare, mobility and sustainability, while the EMFA focuses specifically on opportunities for media such as online offerings and services [199, Recitals 2–7]. These more concrete predictions suggest that legislators see innovation as having clear value for the public, which may justify the subsidies discussed above (§2).

Importantly, the need to balance these predicted benefits with risks to the public and organizations is evident across the reviewed policies. Several frameworks focus on balancing economic growth and competitiveness against protections for specific rights. The GDPR [191, Recitals 2–3] and DGA [198, Recitals 1–5] mention balancing the free flow of data for economic growth with data protection and privacy rights. The Copyright Directive seeks to balance IP rights with technological development [192, Recital 3]. Both the AI Act [200,201] and PLD [202, Recitals 1–5] serve as key consumer protection frameworks to protect the health, safety and other interests of individuals against AI and digital products. The AI Act recognizes specific legal and ethical challenges stemming from bias, opacity, misinformation and environmental sustainability or protection [195, Recitals 27, 61].^62^ The DSA seeks to ensure responsible and diligent provision of online platforms and services while protecting fundamental and individual rights, particularly freedom of expression and non-discrimination [194, Recitals 2–3]. Finally, the PAR echoes this concern with misinformation, focusing on the need to combat voter deception, disinformation and political interference stemming from social media and other digital technologies [204, Recital 4, pp. 7–10].

More generally, the GDPR [191, Recitals 2–3], AI Act [195, Recitals 1, 4–5], DO [106, Recitals 1–2] and EMFA [199, Recitals 2–7] seek a balance between economic growth and technology uptake with fundamental rights and European values broadly conceived. Risks of unfair and distorted competition practices such as lock-in effects, vertical integration and other dependencies feature in both the PLD [202, Recitals 1–5] and DMA, with the latter noting that the economic value created by online platforms and gatekeepers needs to coexist with healthy competition [194, Recital 1].

EU legislators thus envisage a variety of risks to individual rights and public goods such as health, safety, fundamental and individual rights, and consumer protection. At the same time, they believe that innovation will be socially beneficial. Their legislative mandate is to find a balance between these risks and benefits.

### 4.1. The absence of positive obligations

EU tech policy is thus defined by risk mitigation. Reflecting this, the reviewed policies rarely place positive obligations on tech providers and deployers unrelated to mitigation of perceived risks, for example new individual rights or positive equality duties for the public sector [205], [206, p. 727], [207].^63^ Policy rarely prescribes a positive, proactive vision for emerging technologies and the role they can and should play in society. Instead, it tends to be reactive,^64^ seeking to make sense of the novel capabilities of new technologies and their risks, and to create rules to ensure their alignment with fundamental EU rights and values.

This tendency can be found across many of the reviewed frameworks:

— **AI Act**: uses a risk-based approach, where predictive AI systems are categorized according to their predicted level of risk, with different restrictions applying based on degree [190,200], [209, pp. 6–7], [210, pp. 685–686]. Positive obligations, for example to improve education or job satisfaction by deploying AI, are not prescribed. General-purpose AI systems receive similar treatment; specific systemic risks must be mitigated, including discrimination, national security, misinformation and others [195, Recital 110]. Providers and deployers are not, however, required to improve the quality of democracy or national security with AI.
— **GDPR**: uses a risk-based approach [211,212]. Distinguishes between data types based on identifiability and sensitivity to determine their level of riskiness and necessary restrictions on processing. Anonymized data, for example, is lowest risk based on the assumption that a person cannot be re-identified and thus cannot have their privacy violated. Personal data and sensitive personal data warrant comparatively higher degrees of protection [191, Recitals 26, 28, 51, 53, 75–77]. ^65^ The framework does not stipulate that data controllers must use personal data for socially beneficial purposes; rather, they need only to reduce the risk of privacy harms for individuals.
— **DSA**: the DSA sets out requirements for Very Large Online Platforms and Very Large Online Search Engines based on systemic risks posed to fundamental rights, electoral processes, misinformation, gender-based violence and minors, among others [194, Recitals 1–2, Article 35]. In contrast, the DSA does not impose positive obligations related to these risks, for example, to promote fundamental rights or gender equality, or contribute to the mental well-being of minors.
— **Product Liability Directive**: inherently about risk mitigation as a type of tort law. It ensures recourse and redress for individuals harmed by (AI) products, thereby providing an incentive for providers to reduce risks of harm. It does not, however, stipulate that (AI) products need to be socially beneficial.
— **Copyright Directive**: grants IP rights to creators to protect their work. However, it also creates rules, restrictions and time limits to reduce the risk of IP rights stifling further creation by others. The CD does not require creative works to be socially beneficial. ^66^

Defining and measuring the societal benefits of innovation is difficult. A ‘common good’ or positive vision of society towards which AI should be designed and used is not explicitly defined by legislators in the reviewed policies. Provisions and requirements meant to protect individual rights and societal interests from the risks posed by new technologies tend to be more concrete and enforceable than predicted benefits. Policies claim that compliance with EU legal requirements act as a mark of quality for tech products and services, giving them a competitive advantage.^67^ Further, general affirmations of EU rights, laws and values, as well as references to areas such as healthcare, agriculture, food safety and education suggest a vague conception of common good, but details are lacking about how AI (and other emerging technologies) should concretely benefit the public in these areas. Rather than setting specific goals for innovation to deliver concrete benefits to society, laws tend to leave measurement and delivery of predicted benefits open-ended, underspecified and thus unenforceable. This gap means that, at least from a regulatory perspective, the social benefits of new technologies cannot be guaranteed via risk mitigation alone.

### 4.2. Why risk mitigation is insufficient for AI

AI poses a fundamental strategic challenge to EU tech policy because its potential applications and risk profiles are so broad. As a technology that can seemingly replace or augment any type of human intellectual labour, reliably predicting the full range of its possible use cases is practically impossible. The same can be said of the risks it poses to individuals, society, fundamental and individual rights and other interests.^68^

General-purpose and generative AI's risk profile is exceptionally broad in the sense that the range of potential risks grows in step with its applications across sectors [215]. Further, many risks are (i) poorly understood, in the sense that their nature, scale, severity or frequency are not matters of scientific consensus, or (ii) poorly regulated, in the sense that current regulatory frameworks are not effective at mitigating them, do not address them at all or fail to address emerging dimensions of the risk in question.^69^ Examples of poorly understood or poorly regulated risks of general-purpose and generative AI systems include:

— **employment and economic inequality**: widespread unemployment or displacement owing to automation and uncertain economic mechanisms to replace lost labour and income, downward pressure on wages, work quality, collective bargaining power and job security owing to competition from AI for work, homogeneity in AI-driven recruitment decisions [216, p. 221], [217, pp. 254–255], [218, pp. 641, 648–649], [219];
— **education**: cheating, plagiarism, impact on writing, reading, comprehension and information retrieval skills [220–224];
— **skills and competences**: AI literacy, de-skilling, never-skilling and other impacts on professions, standards of practice, automation bias and reliance on AI to accomplish skilled tasks [220–225];
— **technological inequality**: unequal access to AI resources for work, education, personal projects and other areas, and unequal burden of costs from its usage [181,184,226];
— **environment and sustainability**: under-reporting and gamification of efficiency standards, impact on local and regional environment and residents, competition for scarce resources such as water, electricity and land, degradation of local infrastructure [46, pp. 13–15], [203, p. 148], [227–230], [231, pp. 612–613];
— **IP**: unauthorized training and copying of creative works, competition for creative work, devaluing of creative work owing to difficulty of separating human from artificially generated works [232,233], [234, p. 22], [235];
— **public discourse and shared knowledge**: hallucinations, careless speech, deepfakes, AI-generated misinformation and disinformation and algorithmic nudges undermining the quality of public discourse, consensus, shared knowledge and the information environment [71,216], [236, pp. 7–8], [237, pp. 1–2, 11–12];
— **personal security, privacy and integrity**: personalized scams, privacy violations [238–242], deepfakes and misappropriation of one's identity [216, pp. 217–218], [243,244];
— **personal identity and relationships**: impact of sycophancy and parasocial relationships with AI chatbots on well-being, loneliness and mental health [216, pp. 218–219], [245–247].

The breadth and severity of these under-regulated or poorly understood AI risks are stark. EU tech policy is comparatively narrow, meaning it focuses on specific sectors, applications or values and rights and their related tangible risks (e.g. non-discrimination, environmental standards and data protection; §4),^70^ but struggles to effectively manage long-term, holistic and cumulative risks, where the harm is widespread and difficult to measure.^71^ As we have already seen, positive visions and obligations are also conspicuously absent.

### 4.3. Towards positive obligations in tech policy

This situation is unacceptable from the view of policymakers’ mandate to find an appropriate balance between the risks and benefits of new technologies for society. Individuals in the EU are paying substantial subsidies to support AI (§2) and facing poorly understood but wide-ranging risks from its deployment, while only being promised vaguely defined benefits in return (§3). Continuing solely down the pathway of risk mitigation is unsustainable for the future of AI; the costs and risks placed on the public are simply too great, with too little guaranteed in return. Going forward, EU tech policy needs a third goal to rebalance AI governance alongside the goals of ‘risk mitigation’ and ‘economic growth’. We argue that AI providers seeking or currently benefiting from public subsidies should be obligated to provide detailed plans for expected benefits of the technology to individual happiness and social well-being, against which they can later be assessed.

Happiness as a third goal for EU tech policy is justified by two observations. First, AI companies already received many subsidies from and on behalf of the public, in the form of privileged access to resources like land, water, electricity and data; access to public funds and tax breaks; and regulatory leniency and political capture of policy-making processes (§2). These subsidies enable their success on the market but are not given with any guarantees of future societal benefits (but rather only vague promises and predictions by companies and policymakers). Happiness as a policy goal can help to ensure companies actually develop, deploy and govern AI in a way that will concretely benefit the people subsidizing its very existence.

Second, policymakers are obligated to act in the best interests of the public. With AI, they are doing so under conditions of significant uncertainty. The risks discussed above are, if realized, extremely severe and disruptive for society. The predicted benefits are likewise vaguely defined. This raises several questions: why is the only party benefiting from this arrangement AI companies while the other pays the costs? Why are policymakers not compelling companies to concretely define their predicted benefits, and hold them accountable if they do not deliver? The public is in all but name a shareholder of AI companies.

We are not alone in observing the limitations of technology development and policy to deliver promised social benefits. Many initiatives have been founded in recent years to redirect technology development, governance and regulation towards more human-centric, less economic goals.^72^ Scholars have also made influential policy proposals, such as Barbara Prainsack and Alexa Buyx's groundbreaking and compelling proposal to use ‘solidarity’ as a policy and governance goal when regulating data access [190,248,249], Linnet Taylor's work on ‘data justice’ [250], or Jack Balkin and Jonathan Zittrain's proposal to classify data processors as information fiduciaries responsible for realizing the best interests of their data subjects [251,252], [253, pp. 339–341].

Further, using happiness as a public policy goal is also not unprecedented, with many countries already using happiness or the broader concept of ‘well-being’ as guiding principles for public policy decision-making balanced with economic growth or GDP (for an overview of approaches, see table 2 and appendix B). Many of these approaches are based in welfare economics and use theoretical frameworks such as Sen's capabilities approach to operationalize the concept of welfare beyond GDP or market competitiveness [254]. While none of these national approaches listed below are without flaw, and we do not endorse any of them explicitly, their existence shows that happiness already has traction in various forms within policy-making internationally.

**Table 2.** Example happiness and well-being measures.

framework	quality of life				community and environment				material conditions		
Bhutan [273]	psychological well-being	health	education	time use	cultural diversity and resilience	good governance	community vitality	ecological diversity and resilience	living standards		
Stiglitz–Sen–Fitoussi [274, pp. 15, 40–59]		health	education	personal activities including work		political voice and governance	social connections and relationships	environment (present and future conditions)	material living standards (income, consumption and wealth)		physical and economic insecurity
Italy [275, p. 8]	subjective well-being	health	education	work and work–life balance	landscape and cultural heritage	politics and institutions	social relationships	environment	(1) economic well-being (2) quality of services	research and innovation	security
Ireland [276]	subjective well-being	mental and physical health	knowledge / skills and innovation	time use	connections	civic engagement	community and participation	(1) environment (2) climate and biodiversity	(1) housing and built environment (2) income and wealth	work and job quality	safety and security
UK^*a*^	personal well-being	health	education and skills	what we do	our relationships	governance	where we live	the natural environment	(1) personal finance (2) the economy		
OECD [278]	subjective well-being	health	knowledge and skills	work–life balance	social connections	civic engagement		environmental quality	(1) income and wealth (2) housing		safety

^*a*^ These have objective (e.g. inflation and unemployment) and subjective measures (e.g. life satisfaction, perceived happiness and hope), see [277].

In comparison with these approaches and the broader umbrella of welfare economics, here we choose a narrower focus on antecedents of happiness as described in psychology to provide clear, empirical grounding for the concept to serve as a third goal for EU tech policy. This narrower focus enables us to recommend precise interventions in existing EU tech policy to improve individual happiness and social well-being compared with the broader, societal lens of welfare economics. In the following section, we discuss five decades of psychological research on human happiness to understand its antecedents and value for society. Through a series of policy proposals, we then show how happiness can guide technology development, adoption and governance as a counterweight to risk mitigation.

## 5. What makes people happy?

Happiness is not reducible to attainment or a sense of satisfaction in the sense of obtaining what one wants at any given moment. A person may experience an easy life in ways that are disconnected from others, ineffective in practice, or alien to their own values, but these benefits are not expected to last long [255]. In addition, a person may feel stress or disappointment while living well in a fuller psychological sense [256], [257, pp. 150–151], [258, p. 1].

For decades, psychological theories have argued that true happiness is grounded in meaningful engagement in the world [258–263]. Across these traditions, three antecedents of happiness have received extensive empirical support: these are relatedness, self-efficacy (i.e. competence) and autonomy. Framed as basic psychological needs (e.g. SDT) [264], or as fundamental aspects of psychological well-being [261,265,266], these dimensions are understood to be necessary for defining global well-being and predicting sustained happiness.

### 5.1. Relatedness

The first of these—relatedness—reflects the social nature of human beings, who have evolved such that they are fundamentally motivated to form and maintain lasting interpersonal bonds [267,268].^73^ It is not only that relationships matter, but that they matter in a way that facilitates happiness and well-being. A life cannot be understood to be going well if it is marked by chronic loneliness or alienation, even if it feels pleasurable or comfortable.

At its core, relatedness concerns the experience of being connected to others, cared for by them and caring for them [261,270,271]. But relatedness can come in many forms. Relatedness is felt when people feel known, understood and valued, when they experience others as responsive to their needs and perspectives, and when relationships allow for warmth, trust and mutual significance. Research on perceived partner responsiveness, for example, shows that people flourish when they feel that others close to them ‘get’ them, validate them and care about them, suggesting that relatedness is less about the quantity of social connection and more about perceptions of oneself as meaningfully engaged with others [272].

The links between relatedness and happiness have received strong support from large-scale empirical evidence. For example, a recent global analysis reported in the World Happiness Report synthesized data from 22 countries and regions in the Global Flourishing Study and the Gallup World Poll data. It found that stronger social connection and perceived social support were associated with higher life satisfaction both within and across countries [279]. Furthermore, young adults who reported at least one close relationship are 16% more satisfied with their lives on average than those who reported no close ties. This global evidence converges with a separate meta-analysis by Holt-Lunstad *et al.* [280], which synthesized 148 studies and found that stronger social relationships were associated with lower mortality, or in other words, increased likelihood of survival. Together, these findings suggest that relatedness is not just one desirable feature of a good life, but rather that it is a basic condition of well-being.

Crucially, relatedness is primarily, but not exclusively, a social experience. It can also be met through parasocial relationships, including through connection to fictional characters [281] and God [282,283]. The brunt of evidence in parasocial relatedness concerns nature connection, the feeling that one meaningfully connects to the natural world. Connection to nature is particularly relevant in this context owing to the loss of access to land and other natural resources currently being faced by residents living near data centres (§2.1).

Findings show that among people spending time alone in nature, higher connectedness to nature is associated with less loneliness. In contrast, people feel lonelier when alone in nature but feeling low nature connection [284]. The evidence concerning loneliness, in particular, suggests that nature connection can act as a substitute for human connection. Earlier work has also found that nearby nature buffered the harmful effects of low social connectedness on subjective well-being, similarly suggesting that nature may partly compensate for deficits in social ties [285].

Nature connection also appears to matter more broadly for happiness and well-being. Meta-analytic studies report that people who feel more connected to nature report greater positive emotions and life satisfaction [286]. Studies also show that people who report more nature connection report not only feeling happier, but also broader indicators of psychological well-being, including mindfulness, meaning and a greater sense of aliveness [286,287]. Connection to nature is particularly relevant in this context owing to the loss of access to land and other natural resources currently being faced by residents living near data centres (§2.1).

### 5.2. Competence

People are not only social by nature. They are also doers, builders and thinkers. The capacity to act effectively in the world is central to productivity and to happiness [288]. Competence (also termed self-efficacy) refers to the felt experience that one is able to undertake tasks and achieve goals that are felt to be important: that one can act effectively, meet challenges such that efforts put in really matter, learn, solve problems and bring about meaningful and desired outcomes [289]. It is a core psychological resource that shapes whether individuals approach demanding situations with confidence or helplessness [290,291]. It is felt when people take on optimal challenges (challenges that stretch them, but which they can successfully complete) [292, pp. 44–46], [293, p. 90], when they do meaningful or interesting work, and when they receive feedback that signifies progress [294, pp. 256–257], [295].

Competence is understood to be more than a side-benefit of successful actions; rather, it inspires purposeful action [288]. Research on learned helplessness [291,296,297]—the feeling that one's actions are unrelated to meaningful or desired outcomes in the environment—highlights the happiness costs of deficits in competence. People (or, in original research, animals [298]) feel or act depressed when they are repeatedly exposed to uncontrollable outcomes and start to expect that their actions are not related to meaningful outcomes. In these settings, those who expect non-contingency between actions and outcomes stop trying to act even when change has become possible; in other words, helplessness leaves people unmotivated to exert effort and act on the world. These patterns are core symptoms of depression [299, pp. 230–235]. On the other hand, when people feel competent, they see problems as difficult, but they feel motivated to face challenges [300]. Across decades of work on the topic, psychologists have shown that beliefs about one's ability to exercise control over events are central to motivation, persistence and adjustment in major life domains ranging from education to work [290,296,297,301].

People feel happy not when they are presented with no challenges, but rather when they are presented with challenges that feel manageable and worth undertaking. These include better life satisfaction, stronger coping and better adjustment under stress [290,297], [302, p. 60]. At work, experiencing challenging demands is linked to more positive emotions [303, pp. 703–704], so long as they are not excessive and therefore once again undermine competence [304]. When jobs are restructured, those who feel capable of handling the challenge can meet changes with better psychological well-being, stronger engagement and higher job satisfaction over time [305].

Meaningful, rather than frivolous, work also supports happiness through competence. When people experience their work as meaningful, growth-promoting and reflecting their capacities, they are more likely to experience well-being ([289]; see also [306,307]). Research increasingly suggests that such meaningful work is linked to greater happiness across emotional (lower negative emotions), cognitive (better life satisfaction) and physical (i.e. better health) dimensions [308], including in studies that track people's work habits across time [309].

### 5.3 Autonomy

Finally, it is now clear from five decades of research that neither ‘relating’ nor ‘doing’ can foster deep feelings that one is ‘related’ or ‘competent’ without also having the characteristic of being ‘autonomous’ [263]. Autonomy is a fundamentally important, and often misunderstood, driver of happiness. Colloquially, autonomy is often taken to mean independence, self-sufficiency or freedom from the influence of other people; ‘autonomous’ people are assumed to stand apart, resist influence and rely only on themselves. However, this individualistic definition is not how the construct is typically understood in psychology. Researchers understand that one can rely deeply on others and still feel autonomous as in the case of cooperating with doctors’ orders for treatment of a deadly illness, or appear independent while feeling internally controlled as in the case of a hard-working but highly pressured executive whose sense of value as a person depends entirely on his success at work. An SDT conceptualization of autonomy thus differentiates it from both individualism and independence, and in this sense aligns with feminist and indigenous relational-autonomy views, whereby connections with other people, communities and the natural world are not antithetical to autonomy, but rather help to form autonomy-relevant dimensions such as identity, values and self-trust [310,311].

Within SDT and related approaches [267,312–314], autonomy is not understood as separateness or independence *per se*, but instead as volition: the experience of acting with a sense of willingness and inner valuing rather than pressure or compulsion. From these views, people feel autonomous when their actions are endorsed from within, experienced as their own and carried out with a sense of psychological authorship, that is, with the feeling that ‘this is really coming from me’ rather than being imposed from outside (most often by other people, but possibly, other external forces). Autonomy may be felt when one behaves from deeply held values, because actions are meaningful and important, because actions are rewarding, or because acting fulfils curiosity or interest [267]. In contrast, autonomy is undermined when actions are driven primarily by compliance, coercion or felt pressure.

Extensive evidence links autonomy to happiness and happiness-related outcomes. For example, cross-cultural work across four to eight countries at a time suggests that well-being is reliably higher when people pursue goals for reasons they can genuinely stand behind, whereas independence is much less predictive of happiness across cultures [289,315,316]. In a growing number of studies, social relationships are understood to predict well-being only insofar as they fulfil autonomy. For example, in romantic relationships such as marriages, spouses must support their partners’ autonomy to bring them happiness [317], [318, p. 452], and even friendships are based on the mutual exchange of autonomy support, with each partner supporting the agency of the other [319, pp. 314–315], [320,321]. Even a behaviour as fundamentally social as helping others (i.e. prosocial behaviours) conduces happiness benefits for both helper and recipient insofar as they are autonomous rather than pressured or coerced [322,323]. Autonomy, on this view, is not opposed to care; rather, it is one of the conditions that makes care psychologically rewarding.

In one of the largest early studies testing autonomy, Tay and Diener analysed Gallup World Poll data from 123 countries and showed that people who had autonomy experienced more positive emotions and were more satisfied with their lives across the world [321, p. 362]. Importantly, within this massive dataset, autonomy was important above and beyond economic factors such as household income. More recent large-scale evidence again points to global happiness benefits of autonomy. Data from the World Values Survey across 66 countries and nearly 100 000 respondents showed that autonomy was associated with more happiness and life satisfaction across the world, and was an even stronger predictor at the national level than nation-level wealth [324, pp. 12–13].

In sum, this work suggests that it is not enough to experience pleasure and economic attainment. Rather, people feel happy when they are connected to others, pursue and progress meaningful challenges and do both volitionally, from a sense of interest and caring. Relatedness is important for happiness because as social creatures, quality connections outside of the self, rather than alienation, is fundamental to living well. Competence is important because we seek to do well, to build and contribute in meaningful ways. Autonomy matters because we need to operate in a way that expresses and is congruent with what is interesting and valuable, and feel little benefit from either connection or accomplishment when it is felt as pressured or imposed.

## 6. The social value of happiness

Having defined antecedents of happiness, we can turn to a fundamental question that determines the need for such definitions: why should policymakers and the AI industry care that the general public is happy?

First, happiness holds intrinsic value for the public as a central way that people evaluate the quality of their lives [325,326]. The same idea has been posited in economics, which has increasingly recognized that people's reports about a ‘worthwhile life’ centrally involve happiness, and therefore, production or consumption metrics are insufficient alone for defining individual success [327].

This, then, has implications for which benchmarks should be used to identify successful functioning of a society. If society is meant to serve the needs, desires and ambitions of its people, then their own experience of whether life is going well must be considered as an indicator of societal success. Importantly for our purposes, happiness is such an indicator.

But the case for happiness remains strong even if we set aside the point of individual benefits and instead evaluate the utility of happiness as a greater good to society (i.e. social well-being). Happy people are better learners, healthier (and therefore less likely to draw on limited public resources), more productive employees and creative problem-solvers (which can contribute to social progress) and more helpful contributors to a cohesive society.

For example, a large review concluded that happiness is associated with, and often precedes, success across major life domains, including at work [328, pp. 831–832]. Experimental evidence points in the same direction, finding that people who are made happier become more productive in paid tasks [329], challenging the assumption that people need material success to become happy. These findings indicate that happiness is a driver of other socially desired outcomes rather than an end goal achievable through other means. Further, it suggests that the relationship does not only run from productivity to happiness, but rather runs the other way around as well. Happiness helps to bring about many of the outcomes that governments, legislators and industry leaders already value.

A similar pattern appears in education, wherein happiness is positively associated with academic achievement [330, p. 91] and creative performance [331], [332, pp. 1875–1879]. The association between happiness and greater creativity matters because creative thought is the foundation for innovation and problem-solving, including for broader economic and societal advancement.

Finally, happiness also matters because it seems to support prosociality and social cohesion. For example, Gallup World Poll data from 163 countries found that happiness was positively associated with giving time and money to others [333, p. 857]. In other words, happier people are not only more satisfied with their own lives, but they also seem more likely to help others. Arguably, happiness creates a positive feedback loop wherein happier people are more prosocial, which in turn makes them happier, and ultimately, more prosocial [334, p. 232]. In all, a society made up of happier people is likely to be productive, cooperative and caring.

The three psychological antecedents of happiness discussed in the previous section (i.e. competence, relatedness and autonomy) are also important for social well-being as well as individual happiness. Specifically, when people feel competent, they are more likely to approach tasks with confidence, persist when difficulties arise and experience themselves as capable of influencing their environment. When people feel related, they are more likely to trust others, invest in relationships, cooperate and remain embedded in the kinds of stable social bonds that support cohesive social life. When people feel autonomy supported, they are more likely to bring genuine effort, interest and commitment to what they do. As such, these needs are the foundation that allows people to learn, work, create, care and participate well in families, schools, workplaces and communities, ultimately leading to stronger and more productive communities [264,335].

Indeed, one reason that we have focused on these three needs in this article is that each supports a different aspect of human behaviour which ultimately contributes to social well-being. Competence helps sustain mastery and persistence, particularly but not solely in the face of challenge. Relatedness builds mutual care and belonging, which in turn support trust and cooperation and ultimately, social coherence. Autonomy energizes action; research shows that people are more engaged when what they do feels self-endorsed [336, p. 1025]. A society that supports these conditions is therefore not only making people feel better. It is also creating the motivational and relational conditions under which people are more likely to contribute and cooperate [271], [336, p. 1025], [337].

Happiness therefore should not be dismissed as a soft or weak policy target. It is both an end in itself, and a means to other, clearly endorsed, ends.

## 7. Happiness as a third goal for EU tech policy

Current EU tech policy attempts to balance the risks and benefits of emerging technologies measured in terms of economic growth and risks to fundamental and individual rights and EU values (§4). AI industry promotional material likewise focuses predominantly on the economic benefits of their technologies (§3). These dual trends have resulted in a situation where the subsidies offered on behalf of the public to the AI industry are not offering a fair ‘return on investment’ to society. A more promising approach could be to use ‘happiness’ as a policy mandate and metric to evaluate the predicted and actual risks and benefits of innovation.

Psychological research offers empirical evidence about what actually makes people happy (§5). According to SDT, the key antecedents of happiness are relatedness, competence and autonomy. *Relatedness* refers to the core psychological need of having (social) connections with friends, family, community and nature. *Competence* refers to the drive to understand, learn, master and use skills to meet meaningful goals. Finally, *autonomy* refers to a sense of willingness to pursue goals. These three key elements are the foundation for predicting happiness and well-being.

This ‘happiness framework’ can work as a compass for tech policy decisions. Evidence is mounting that emerging tech products and services can harm psychological well-being and happiness. The three antecedents of happiness cast emerging use cases, risks and benefits in a new light that suggests different policy priorities and goals are urgently needed to strike an appropriate balance between growth, public goods and fundamental and individual rights. A few illustrative examples show their value for understanding emerging tech policy challenges.

*Competence* is highly relevant to risks of deskilling via generative AI. Recent deployments of generative AI across universities and schools may undermine students' educational skills. Evidence is rising that generative AI harms students’ capacities in information retention,^74^ performance on mathematical reasoning and reading comprehension tasks [222,339], writing skills [340], critical thinking [341, p. 15] and creativity [342]. Similar phenomena can be observed in medicine with deskilling of doctors from the use of AI tools; one study showed, for example, that doctors are less able to detect cancer manually after having used AI tools for only three months [343]. Cognitive abilities are inherently and instrumentally valuable to society (e.g. having competent lawyers and doctors), but also strong antecedents of happiness.^75^

*Relatedness* is uniquely poised to make sense of the impact of AI chatbots. Companion chatbots for friendships, relationships and grieving are increasingly common [344]. The current business model—very similar to the attention economy [345, pp. 12–16]—focuses on keeping users engaged for as long as possible [71,346,347]. Chatbots that are designed to mimic human emotion and empathy enable parasocial relationships (§5.1), but reduce opportunities for real human and nature connection [344]. Engagement with chatbots can harm mental health and lead to ‘AI psychosis’ [348], suicide [349–351], loneliness, dependence and isolation [339,352–354]. Finally, data centres need natural resources such as land, water and electricity [46, pp. 13–15], [203,227,228], and contribute to climate change [355, pp. 2–3]. People living near data centres are losing access to natural land and resources to subsidize their construction and operation, further limiting opportunities for connection to nature [356, p. 22].

*Autonomy* illuminates the risk of large language models (LLMs) and chatbots turning increasingly sycophantic to keep users engaged and online, as well as other novel forms of persuasion ([245,357]; see also [358]). Personalized responses can very effectively nudge users, for example to buy products or services [359]. These trends, as well as decisions to incorporate advertising into generative AI, will probably reduce people's autonomy, or ability to willingly pursue meaningful goals without coercion or undue external influence [242,360].

### 7.1. Policy proposals

Policymakers and regulators can use the proposed happiness framework, with its three key elements, to balance societal and industrial interests in future tech policy. It can also help industry and the public make decisions about the development, deployment, adoption and governance of emerging technologies. We propose several concrete suggestions to incorporate these ideas into current tech policy-making, business practices and public adoption.

#### 7.1.1. Happiness to receive public subsidies

AI companies currently enjoy significant public subsidies to enable their research and development (§2). When policymakers are deciding whether these privileges should be granted or extended, they should require companies to provide detailed plans describing expected benefits for individual happiness and social well-being. These plans should ideally include design specifications and design choice justifications, specific planned use cases, happiness metrics and scientific evidence underpinning all of the above. Developing specific, binding evidentiary requirements, including, for example, documentary or technical standards, should be the subject of future research and policy development. This work would be ideally carried out by independent bodies or regulators themselves to avoid dilution via lobbying in practice [114,361].

It should be noted that our proposal finds common ground with prior work on public value and innovation, such as Prainsack's notions of data justice and solidarity in the realm of data governance.^76^ This can introduce new incentives beyond profitability, thereby encouraging companies to change how they design and deploy new technologies. It likewise provides additional leverage to regulators and the judiciary to demand changes when harmful subsidized products are placed on the market (see below).

#### 7.1.2. Happiness as a third goal of EU tech policy to ensure socially beneficial AI

Policymakers and regulators have a legal mandate to balance competing interests (§4). The vague promise that AI products and services will be socially beneficial is used to justify lobbying, deregulation and weakening of fundamental and individual rights protections for the sake of innovation. Here, happiness can also serve as a balancing factor within the strong risk mitigation approach taken in EU tech policy. The current approach predominantly takes a narrow focus on reducing risks rather than ensuring concrete, positive impacts from AI products and services. Happiness can create a ‘floor’ for policy-making to ensure AI is socially beneficial, not only less risky. The fact that a technology is less harmful does not mean it is socially beneficial.

#### 7.1.3. Enforcement and penalties

When administering penalties, regulators and judges must take individual circumstances into consideration. For example, small and medium-sized enterprises (SMEs) traditionally receive lower fines than multi-national companies. Illegal actions committed with malice or intent are punished more severely than those resulting from negligence. If technologies harm people's happiness measured in terms of relatedness, competence or autonomy, penalties should reflect this fact. Regulators and judges could, for example, require changes to the design of a product to mitigate specific harms to these three antecedents of happiness. AI data centre operators found to have misreported resource usage or environmental impact, for example, could face steeper fines owing to the downstream impact their actions have on residents' access to nature (§5.1).

#### 7.1.4. User literacy, choice and autonomy

User empowerment, choice and literacy are important factors in people making decisions about how and to what extent they want to integrate technology into their lives. The three antecedents of happiness can help end users make these difficult decisions. Through public awareness campaigns and knowledge transfer, people can be encouraged to ask themselves questions like: will this technology enable me to make closer social or nature connections? Will it help me to master a new skill, and does it require dependency (e.g. generative AI)? Will it help me form and pursue life goals? Overall, will it make me happier?

#### 7.1.5. Happiness through product design

The antecedents of happiness can help the AI industry. Relatedness, competence and autonomy can guide how products and services are designed and deployed, and serve as a counterweight to economic incentives and socially harmful business models. Companies should focus more on developing products that promote these three concepts, albeit without taking a simplistic or paternalistic approach (e.g. forcing users of a social networking app to contact friends once a day to improve relatedness).^77^ Technologies are, of course, already designed with them in mind, but are often backed by harmful business models or features that outweigh their benefits to happiness. For example, dating and friendship apps enable human connection, digital libraries and online lectures enable learning and mastery of new skills, and health tracking apps enable people to achieve their health or life goals. Each of these examples coexists with socially harmful design choices meant to maximize profit or engagement at the cost of user well-being, such as ‘swiping’ behaviour on dating apps or excessive gamification of health goals.

#### 7.1.6. Happiness curricula

People often struggle to understand and predict what makes them happy. Offloading cognitive work by using an LLM to write an essay for school may appear immediately beneficial to a stressed student. Connecting with a chatbot to alleviate loneliness might also seem like a good replacement for human connection. However, people require support to make sensible choices in such scenarios that will lead to more fulfilled lives.

Happiness research can provide such support if embedded in school curricula, AI literacy obligations, or similar learning opportunities. Some countries have already invested in this direction; Delhi, India, for example, implemented the Happiness Curriculum in 2018, which focuses on holistic learning aims rather than strictly academic outcomes [363].^78^ In Germany, a research project at TU Braunschweig advocates for integration of daily ‘happiness lessons’ into the curriculum [365]. While not a formal subject in school, the UK also urges investments in resources for well-being and mental health for students, and expects schools to cover mental health issues as part of the relationships and sex education and health education curriculum [366]. Based on our findings, we recommend further rollout of such initiatives with a specific focus on the role of emerging technologies in happiness and how to make sensible decisions about personal use.

These proposals are ambitious and open to future interpretation. Many key questions of implementation, measurement and enforcement are intentionally left open for future work.^79^ While fully developing and justifying the necessary evidentiary, policy, organizational and technical requirements to implement and enforce these proposals in practice goes beyond the scope of this article, together they provide an initial direction for future research and policy-making efforts to rebalance EU tech policy in favour of social well-being.

## 8. Conclusion

Much like the legend of Perseus, the myth of self-made tech billionaires collapses under closer scrutiny. Whereas Perseus required divine gifts to slay Medusa, modern AI companies benefit from the substantial public subsidies lavished upon them by adoring governments. Perseus smartly honoured his divine patrons in thanks for their assistance, yet the same cannot be said of companies benefiting from the people underwriting the current AI boom. This technology may indeed lead to the unfettered economic growth and efficiency predicted by companies, but its impact on society, individual rights and well-being, employment, the environment and other public goods remains far more uncertain and looks increasingly harmful. The public seems to be left blindly subsidizing an international AI ‘arms race’ which will, at best, indirectly benefit them, and at worst actively harm them. Just like the heroes of ancient Greece, AI companies should fear losing ‘divine’ favour if their benefactors feel they have been taken for granted.

The public is in all but name a shareholder of AI companies.^80^ To re-establish an appropriate balance between economic and social benefits in EU tech policy, we have proposed a new mandate and metric: happiness. Our proposal is an attempt to rebalance the focus of regulators from predominantly economic considerations towards a more holistic idea of societal progress. Being happy is a goal in itself, but happy people are also good for society. Happiness allows people to learn, work, create, care and participate well in families, schools, workplaces and communities. They are better learners, employees, problem-solvers, more helpful people and better able to build strong social relationships [328, p. 825]. Happiness is a driver of many of the outcomes that governments, legislators and industry leaders already value.

Policymakers should increasingly prioritize relatedness, competence and autonomy as antecedents of happiness to ensure that public support goes to innovation that genuinely improves human happiness and well-being. These goals can be equally beneficial for companies and users themselves, helping guide better decisions which deliver economic and social benefits in tandem, rather than at the expense of one another. Making technology less harmful does not mean it is socially beneficial on balance. The AI industry must be compelled to aim higher, to actively enhance human well-being and fundamental and individual rights with innovation, rather than merely erode them less than originally projected.

## Figures and Tables

**Table 3.** Reviewed AI industry documents.

company	source link and title	target audience	purpose	use cases (application domain and/or tasks)
**IBM**	Generative AI + ML for the enterprise https://www.ibm.com/account/reg/us-en/signup?formid=urx-52356	businesses	Streamline generative AI adoption around guiding elements on openness, targeted implementation, reliability and empowerment.	Generative AI and ML models in content moderation, personalization, anomaly detection, forecasting and data augmentation.
	What can AI and generative AI do for governments? https://www.ibm.com/think/topics/generative-ai-for-government	public sector (governments and citizens)	Providing background driving AI investment in the USA—facilitated by research initiatives driven by the previous US Biden Administration—and providing examples of government agencies adopting AI solutions to identify the value of large generative AI models.	Examples on AI initiatives at various stages of US government: tax returns, global health supply chain, citizen engagement in the area of public services and immigration and health services. Notes three areas for potential investments for AI solutions pertaining to the work and labour at different levels of government, citizens engagement and support as well as modernizing legacy systems at government.
	AI in Action 2024 https://www.ibm.com/think/reports/ai-in-action	businesses	Distil distinguishing features between ‘leaders’ and ‘learners’ in generative AI adoption, usage and profitability.	Prioritization of use cases for leaders to improve customer experience, IT operations and automation, assistance and AI-search engines for internal and external applications and cybersecurity.
	5 Trends for 2025 – ignite innovation with people-powered AI https://www.ibm.com/downloads/documents/us-en/11630e2b96302ccc	businesses	Underlines a shift from generative AI to agentic AI applications and notes opportunities that go hand in hand with this trend. Surveyed organizations to identify opportunities and hurdles in adoption.	Main prioritization for businesses identified in this survey, this entails task-delegation of ‘leaders’ to employees, general workflow optimization and supply chain and business effectiveness.
**Google Cloud**	The ROI of Gen AI: a global survey of enterprise adoption and value https://services.google.com/fh/files/misc/the_roi_of_generative_ai.pdf	businesses	Surveys the financial impact and benefits organizations report based on the adoption of generative AI and the financial impact, as well as business value of generative AI in terms of return on investment (ROI) increases, productivity gains, business growth and increased user engagement.	Enumerated examples of user engagement in specific sectors are retail and supply chain, financial services, healthcare and life sciences, and manufacturing and automotive industry.
	2025 public sector report: state of AI infrastructure https://services.google.com/fh/files/misc/google_cloud_state_of_ai_infra_public_sector.pdf	businesses	Surveyed businesses and individuals in leadership positions in companies across industries to identify main priorities, concerns and extent of generative AI adoption and infrastructure investments.	Notes widespread Generative AI adoption and production in tech-related industries, financial services and manufacturing. Key use cases are data analysis, automation of internal processes and customer-facing tasks.
	1302 real-world gen AI use cases from the world's leading organizations https://cloud.google.com/transform/101-real-world-generative-ai-use-cases-from-industry-leaders	businesses and public sector	Updates findings of businesses and public sector adopting Google agentic AI solutions; summarizes five top insights and trends that reveal a shift from AI assistant towards using agentic teams and multimodal systems, using AI for legacy systems in cybersecurity, media creation and personalized marketing.	Outlined list contains use cases of agentic AI in in various Industry and public sector segments including automotive and logistics, business and professional services, financial services, healthcare and life sciences, hospitality and travel, manufacturing and industrial electronics, media, marketing and gaming, the public sector and non-profits, retail and technology industry.
	Welcome to Google Cloud Next ‘24 https://cloud.google.com/blog/topics/google-cloud-next/welcome-to-google-cloud-next24	businesses	Outline of expanding partnerships using Google Cloud for agentic AI and further development of scaling up AI infrastructure.	Examples of how agentic AI supports opportunities in retail personalization, healthcare and business transactions. Further examples underscored in relation of agentic AI adoption include cybersecurity, workflow optimization, customer care, coding support and generation, secure content creation, digital watermarking and supply chain management in different industries.
**Google**	Agents https://www.kaggle.com/whitepaper-agents	businesses (i.e. developers)	Outline of components in reasoning, logic and external access in Generative AI agents. Exemplification of Google extensions, functions and data stores to interact with Google models.	Focus is on functionalities, including linking models with features that allow better contextual interaction with external systems, enhances model performance with specific knowledge.
	AI agents companion https://drive.google.com/file/d/1GVPdwEh48bErTNdhxD0vqxPAifSx1I6Y/view	businesses (i.e. developers and servers)	Outline of agent evaluation methods, Google agents products and implementations, as well as links evaluation with assessing ROI potential.	Presenting the value of Google agentic AI products to measure enterprise productivity, synthesize information and enhance search including information retrieval. Includes a case study on the application of Google agentic AI products in tasks relevant to the automotive sector and scientific research.
	AI works for governments: a digital sprinters report https://www.pwc.com/gx/en/ai-services/ai-works-for-governments.pdf	public sector and general public	Empirical outlook estimating the potential productivity gains and other economic impacts of AI adoption in the public sector. Focus is on top-down and bottom-up quantitative analysis in selected countries in Latin America, Middle East and Africa.	Provides examples of planned or ongoing AI projects to underscore economic benefits connected by three pillars which are: (i) fiscal management and efficiency, (ii) public service delivery improvement, and (iii) GDP growth and increases in household income and productivity (p. 7). Key domains are: disease detection, supporting farming programmes, agricultural workflows, information retrieval in government services, supply chain transparency, flood forecasting, infrastructure planning, supporting outreach and education, airport operations, and the tourism sector.
**DeepMind**	AlphaFold: five years of impact https://deepmind.google/blog/alphafold-five-years-of-impact	public institutions and general public (i.e. science and research)	Providing proof on increased adoption of AlphaFold models in scientific research.	Outlines AlphaFold models’ adoption in biological research and underscores potential for other use cases, including scientific discovery as a whole, as well as fusion and Earth sciences.
**Microsoft**	The business opportunity of AI https://news.microsoft.com/source/wp-content/uploads/2023/11/US51315823-IG-ADA.pdf	businesses	Examines how ‘leading’ organizations adopt generative AI, their benefits and some challenging to overcome for successful generative AI integration.	Key use cases include the automation of organizational, workflow processes and IT, cybersecurity and fraud detection.
	Transforming public sector services using generative AI: global case studies https://wwps.microsoft.com/wp-content/uploads/2024/02/Transforming-Public-Sector-Services-Generative-AI-Report.pdf	public sector (i.e. organizations departments, services)	Outlines a case study approach to the different benefits and gains of generative AI integration in the public sector.	Outline of various ongoing projects on Generative AI adoption various levels of government internationally, such as in the housing sector, optimizing administrative processes and supporting citizens’ digital interactions.
**OpenAI**	Identifying and scaling AI use cases https://cdn.openai.com/business-guides-and-resources/identifying-and-scaling-ai-use-cases.pdf	businesses	Providing guidelines (i.e. key principles, use cases, checklists and examples) for businesses to identify and scale AI use cases and a framework to prioritize projects of high ROI/low effort values.	Examples of use cases include: content creation, research, coding, data/analysis, ideation and strategy, automation along key challenges to address ‘repetition and low value tasks’, address ‘skill bottlenecks’ and ‘navigating ambiguity’ (p. 6).
	Introducing OpenAI for government https://openai.com/global-affairs/introducing-openai-for-government/	public sector (i.e. public servants)	Outlining the OpenAI initiative for their products’ adoption at the US government. Details how OpenAI supports US government by giving access, providing support and custom models.	Enumerated examples on optimizing workloads of civil servants and improving service delivery on federal level. Some further examples could include supporting the Department of Defense streamlining administrative operations in the areas of cyber defence and healthcare services access and data analysis.
**Amazon Web Services**	The generative AI advantage: a leader's guide to using your data as a differentiator https://d1.awsstatic.com/psc-digital/2024/gc-600/genai-adv-tdm/The-generative-AI-advantage.pdf	businesses	Details a ‘data strategy’ for businesses and ‘leaders’ to secure a ‘business value’ in generative AI adoption. This strategy is supporting model customization, data quality, security, human oversight and governance, and that is embedded in organizational processes and businesses’ ‘mindset’ (p. 14).	Outlines examples of AWS collaboration with banks, marketing corporations and industry where data-driven decision-making, machine learning and generative AI where relevant for prioritizing use cases in fraud detection, marketing communications, advertising campaign development, administrative task automation, clinical decision-support, coding support, data analysis and governance.
	A year of progress: AWS update on the $50 million generative AI impact initiative for public sector https://aws.amazon.com/blogs/publicsector/a-year-of-progress-aws-update-on-the-50-million-generative-ai-impact-initiative-for-public-sector	public sector	Sharing insights from the Amazon Web Services (AWS) Impact investment and supporting organizations and initiative collaborating with the public sector and other partners in Industry and giving cloud credits, training and technical skills support for projects.	Outlining some implementations of AWS technologies in the public sector, including healthcare, education and government services.
**Nvidia**	Demystifying generative AI https://www.nvidia.com/en-us/data-center/demystifying-generative-ai/	businesses	Outline of platforms for different generative AI applications with the background to ensure fast market-entry and return of investment.	Top impact areas of Generative AI use cases in next 18 months: ‘software development, product development/design and customer engagement’ (p. 1).
	AI-ready enterprise platform, public sector https://resources.nvidia.com/en-us-global-public-sector/public-sector-solution-platform	public sector	Presenting the NVIDIA AI Enterprise software suite for government agencies to support AI solutions and create ‘business value’ from AI (p. 1).	Notes the integration of AI within different systems, including technologies for infrastructure development, spatial modelling, building and information modelling of equipment, new tools and systems for managing citizens data, AI techniques for supporting citizen engagement and communication, object detection and for decision-making as well as required infrastructure to support development and deployment of AI systems.
	How can generative AI transform the public sector? https://resources.nvidia.com/en-us-global-public-sector/generative-ai-nvidia-dgx	public sector	Introducing Nvidia suites and solutions to streamline generative AI adoption in the public sector.	Introduction of different Nivida suites and solutions to enable the public sector customization and running of generative AI models without mentioning a specific application. Overall aims are speeding up AI deployment with foundation models and securing an ‘accelerated infrastructure’ (p. 2) while ensuring security considerations and lower power computation.
**Anthropic**	Building trusted AI in the enterprise https://assets.anthropic.com/m/66daaa23018ab0fd/original/Anthropic-enterprise-ebook-digital.pdf	business	Sharing insights and examples from Claude and Anthropic AI assistant implementations and different stages for successful generative AI integration.	Example use cases include supporting customer experience, increasing team ‘productivity and creativity’, optimization of processes as well as domains in fraud detection, code generation, customer support, ‘ticket routing’ and data analysis.
	The Anthropic economic index report: uneven geographic and enterprise AI Adoption https://www-cdn.anthropic.com/7b76335c444876a93fa22a63aabb4aeb820aff25.pdf	businesses	Examining Claude.ai conversations and enterprise API usage to identify patterns and shifts in ‘frontier AI’ adoption in different regions and using data from August 2025. Outlines the ‘Anthropic AI Usage Index (AUI)’ (p. 3) to reveal that Claude.ai use is correlated with income, local economy factors, degree of adoption, usage patterns within the USA and globally.	Patterns in Claude.ai conversations reveal a shift in increased AI usage in ‘knowledge-intensive fields’ (i.e. education and science). Claude.ai conversations indicate changes in the nature of task entailing increased AI for electronic sources and database search, creation of instructional materials by educators, code creation and a greater delegation of tasks to AI.

**Table 4.** List of predicted and observed benefits in reviewed AI industry documents.

	promised benefits	
company and document title	observed benefits	projected benefits
**IBM** generative AI and ML for the enterprise	IBM product adoption increased accuracy time saving accelerate model production	creation of a competitive advantage achieve productivity gains ‘enhance outcomes’ (p. 3) ‘fast-track innovation at scale’ (p. 3)
**IBM** What can AI and generative AI do for governments?	modernization of processes improve efficiency improve citizen engagement advance public health initiatives	worker upskilling and hiring, reduce operational cost personalizing and streamlining citizen services, migrating legacy technology systems
**IBM** AI in action	improvement of revenue rate (significant and moderate) improvement in profit margins (significant and moderate) value use cases: cybersecurity and resilience; customer satisfaction, engagement and retention; marketing effectiveness; streamlined processes and staff productivity	competitive advantage (through data management, ‘high-quality and propriety data’)
**IBM** 5 trends for 2025—ignite innovation with people-powered AI	ROI increase from hybrid-architectures competitive edge through Gen AI use in idea generation faster response to ‘market shifts’ (p. 23)	‘benefits from generative AI will exceed potential risks’ (p. 8) productivity gains from ‘upskilling and reskilling’ (p. 12) ‘proactive’ mitigation of climate and geopolitical impacts (p. 16) reducing ‘development costs’ (p. 20) ‘business model, production and service innovation’ (p. 23) ‘product differentiation’ (p. 23) ensure ‘product trust’ and ‘product quality’ (p. 23)
**Google Cloud** the ROI of generative AI	fast ROI increases annual revenue increase revenue growth increased employee productivity business growth improved user experience and engagement increased revenue estimate improved identification and response time to security threats	sustained ROI increase (immediate and long-term/ 5+ years) competitive ROI edge and differentiation in marketing, back-office workflows, productivity and roll out of new use cases adaptivity to market demands owing to shorter innovation cycles ‘greater returns’ owing to immediate and long-term investment in AI (p. 31) near-term effects: ‘improved profit margins and accelerated innovation’ (p. 33) long-term effects (media and entertainment and telecom industry, retail and supply chain and health and life sciences): ‘greater market share’ and ‘competitiveness’, ‘improved customer experience’, ‘strategic decision-making’ and ‘employee productivity’ (p. 35)
**Google Cloud** state of AI infrastructure	improved customer experience organizational productivity improvement optimized operational costs	ability to ‘compete’ in an ‘AI-driven future’ (p. 8) top ROI gains in increased ‘employee productivity’, ‘customer satisfaction’, optimization of workflows and internal processes (p. 24) other ROI gains in ‘competition’, ‘market share’, ‘revenue growth’, ‘sales and revenue’ and reduced ‘operational costs’ (p. 24)
**Google Cloud** 1302 real-world gen AI use cases from the world's leading organizations	value for automotive and logistics industry: efficient operational saving for task completion, customer engagement, reduction of workload responding to customer enquiries, revenue and customer portfolio increases, real-time advertising, workflow optimization and transparency, reduction of duplicate enquiries, accelerate production cycles at reduced costs, work-time reduction, improved data access with reduced manual reporting and ‘increased delivery effectiveness’, supply chain transparency and regulatory compliance	value for automotive and logistics industry: ‘safe, efficient and user-focused’ solutions, real-time risk prevention, efficient inventory planning, making vehicles ‘safer, more sustainable, and more efficient’, reduce manual processes, reduce time and increase accuracy in inventory and warehouse management and tracking, employee productivity, efficient resource allocation in marketing, work efficiency and productivity, efficient and optimized software development, accelerate industrial planning, data analytics for safe and sustainable transport management and packaging tracking
	value for business and professional services: debt collection efficiency and reduction of operational costs; faster service care; reduced time for administrative processes and ‘recruiter efficiency and reduction in technical debt’; accelerate accurate document analysis; accelerate project processes and document drafting and accuracy in data extraction in legal documents; reduce repetition in legal processes; increased accuracy and reduced response time to customer enquiries; increase campaign reach; save time in legal drafting; productivity gains and cost reduction along operation, sales and service; reduced turnout time; reduced campaigning costs and time; increased e-commerce top-position listings, daily sales and revenue; reduced workflow, productivity gains in marketing teams and enhanced customer engagement; faster adoption and more efficient client delivery; ‘workload gains’ in coding; productivity improvements in work-tasks and handling enquiries; compressing manual tasks; time reduction in content generation; scaling up and streamline management of customer bases; ‘cost-per-acquisition’ gains; reduced research and content creation time; overall productivity gains and lowering operational costs; accelerated onboarding; increased speed in security remediation and reducing approval times value for financial services: time reduction in credit approval; specialized customer management; productivity increases and faster call resolution; enhanced usability; instant personalization; saving time through automation of repetitive tasks; accelerating research time and decision-making; internal employee productivity gains; increases in quality and quantity of work performed at shorter time; faster email drafting; more accurate decision-making; lower operational costs; streamline tax assessment processes; accelerated data processing, data analysis, generation of financial models and processing of transactions; elimination of service interruptions; higher match rates with large-scale processing and improved accuracy; increased profit, accuracy and speed in fraud detection and resource cutting on technical skills	value for business and professional services: customer satisfaction; employee satisfaction; ‘enhanced ecommerce experience’ through faster ‘order-to-cash’ processes, personalized monitoring and customer care; replacing manual processes in sustainability intelligence, save time and increase accuracy from manual work and reduction of costs for customers; streamline business ownership; management of diverse application domains; secure information management; reducing internal research time and faster employee support; optimized internal workflow processes; re-focus employees to focus on ‘strategic’ and ‘creative’ tasks; reduction in bottlenecks on cost identification; maximize ROI and client ROI in hybrid-teams; time reduction in manual document review; minimize errors in legal drafting; a more diverse and inclusive work culture; increased internal knowledge sharing; faster production cycle; enhancing retail customer sales; quicker response time to emergencies; faster information insights; increased accuracy in search and proactive risk identification in legal compliance and audits value for financial services: ‘streamlining regulatory compliance’ and improved decision-making, optimized and improved customer engagement and satisfaction, increased and more accurate personalization, efficient internal collaboration; faster and improved accuracy in risk analysis and reduction, more time-efficient customer service; creative problem-solving for clients, streamline software development, secure validation of financial information, faster HR and sales response times, faster and accurate processing of loan applications, faster meeting of deadlines, enhanced decision-making in risk prioritization and faster response time to security threats value for healthcare and life sciences: streamline evidence-based decision-making and personnel and patient management, enhanced resource allocation, improve accessibility, faster disease detection, enhanced patient monitoring, optimized clinical workflows, informed clinical decision-making, identify health trends, speed up accessibility to new medication, streamline patient experience in clinical trials, improved customer and
	value for healthcare and life sciences: personalized guidance and user support in wellness and disease self-management; increased accuracy in patient self-management; cost-effective and efficient scaling of resources; reduced employee onboarding time; faster expertise delivery; faster and accurate regulatory compliance checks and saving costs; increased employee productivity; faster, more efficient and revenue positive task-completion; more accurate diagnosis; reduction of administrative load; faster delivery of health services; faster information searches and reduction of cases requiring escalation in customer service; reduced time spent on denial appeals; reduced infrastructure costs and increased annual savings; accelerate discovery processes and cutting costs and accurate image verification for marketing value for hospitality and travel: improved API load time and platform latency, increasing reservation conversation rates and increased restaurant search visibility, content personalization at scale, lower cost and reduced time in marketing and campaigning content creation, increased marketing revenue, reduced new-arrival enquiries, saving employee time, increased ad revenue and productivity in ad teams, optimized airline processes at reduced cost and reduction of manual security risk classification value for manufacturing and Industrial electronics: automatic summarization at reduced query time, reduced audit costs at faster pace and increased accuracy, accelerate development cycle and enhanced manual data analysis, reduced customer response times, faster quote generation, workforce automation with increased quality and reduced costs on customer services, performance improvements owing to technology migration with accelerated backups and reduced time in business planning, fraud reduction, faster data querying, streamline document search and faster workflow deployment without coding skills, accelerating internal communication and collaboration, increased operational efficiency, reduced data query times, reduction of service interruptions, increased market forecasting accuracy and waste reduction	employee experience, reduced query times, reducing clinical documentation tasks and free up time for patient-centred care, saving ‘lives’ by streamlining donor search and blood supply, reducing human intervention, accelerate research and development, streamline administrative work, improved health outcomes, enhanced healthcare management and follow-up treatments, efficient healthcare planning, accelerate disease screening, speed up processing on medical orders, increased ROI and accelerate drug discovery and biomedical research value for hospitality and travel: faster personalization with decreased costs, enhanced customer experience and satisfaction, reduced query times, greater employee collaboration and productivity, reducing task frictions and enhanced brainstorming of new content ideas value for manufacturing and industrial electronics: users ‘unlocking new levels of productivity, creativity, and enjoyment’, personalized customer experiences with different modalities, tailored product recommendations and advice, supporting the development of innovative, sustainable solutions in agriculture, improve operational efficiency, accelerate employee productivity, streamline organizational tasks and free up time for team building, enhance decision-making, reduced carbon emissions, optimized energy management, reducing greenhouse gas emissions, employee productivity and efficiency increases owing to automation of manual and ‘time-consuming’ tasks, faster scaling of start-ups’ sustainability innovations, improved infrastructure planning and climate change resilience and enhanced supply-chain transparency and data access value for media, marketing and gaming: maximize ROI of ad spending; enhanced customer engagement with increased monetization; increased personalization; enhanced targeting; accurate, reliable and trustworthy information search; supporting user sports literacy; enhanced immersion with content; reduction of manual labour in locating content; real-time optimization and monetization of user engagement; improved customer
	value for media, marketing and gaming: optimized content discovery; increased news search and site engagement; reduction of internal, manual tasks; increase in ‘campaign Return on Ad Spend’; faster ad generation, more accurate ad call identification and reduction of time in analysing industry trends; support article editing; infrastructure cost reduction and reduced developer time investment; faster task completion; significant cost reduction in content generation; shorter production cycles and saving media storage; rise of incremental sales with ad engagement; boosting user engagement and retention in gaming and supporting ‘gross merchandise value’ value for public sector and non-profits: streamline and secure better citizen connection with healthcare services, enhanced student learning support, personalized student-learning, faster assistance on public services, reduce response times to citizen enquiries, supporting transparent communication with citizens, supporting accessible student career guidance, time reduction in drafting action plans, productivity gains in administrative and judicial processes, free up time for client education, increase employee productivity and optimization of operational and administrative processes in telecommunication value for retail: increases in customer carts addition and reduced page load, customer satisfaction, faster and efficient product discovery, increased site visits and sales, faster response-rate to consumer enquiries, revenue increases, reduced ‘null searches’, increased sustainability, improved customer recommendations, higher conversion rate, increase in ‘unique’ product sales, increase of listing ‘completion rates’ at reduced time, reducing workload pressure, reduced manual tasks, securing employee security, increase in average purchases, increased sustainability, employee satisfaction, enhancing work accuracy and increase return on investment value for technology: improved ‘cost efficiency’, reduced ownership costs, reduced operational costs and reduced customer support costs, raised sales conversion	retention; free up time on manual tasks to engage with other stakeholders; supporting employee brainstorming and ideation; supporting manuscript evaluation and enhanced auditing its content; inclusivity and access to different media; efficient scaling of advertising campaigns and optimizing workflows; ROI business gains owing to social media engagement; making content creation accessible to businesses and optimizing content creator search for brand campaigns value for public sector and non-profits: simplify US immigration and attract talent, support sustainable behaviour, inclusive support for job seekers in their career development, speed up claim appeals, ensuring efficiency and accessibility of transport services, streamline hiring process, personalize and optimize public service offerings, accelerate grant writing and supporting researchers from under-represented communities, streamline non-profit work with limited resources and personnel, faster research and information gathering and enhance fact-checking and misinformation value for retail: increased personalized interactions, guiding informed purchasing decisions, ‘customer trust’, ‘faster and seamless customer experience’, operational efficiency, enhanced brand loyalty, workload efficiency, significant ROI increase, optimize workload and ‘pressure’ of customer support teams, ‘supercharge creativity’, streamline ‘knowledge-discovery’, productivity increase, enhanced and efficient decision-making and increased focus on ‘high-level analysis’ value for technology: faster problem-solving and response time to customer enquiries, improved customer experience, inclusive and accessible education, increased work productivity, support ROI, enhanced customer personalization, support customer engagement, accelerate information-retrieval, supporting the solving ‘complex business problems’, supporting knowledge-discovery and personal organization, supporting faster and efficient information-sharing across teams, efficient task-execution, enhanced cybersecurity protection, waste reduction, improved response to customer needs,
	rates, optimized processes, reduced AI model costs, increase in transaction traffic, increased user engagement, accelerate product development at reduced costs, accelerate incident investigation, reduced ‘technical debt’, increased business revenue and candidate retention, boosting online education productivity, accelerate customized development, increased employee satisfaction, optimized educational site operation and enhance productive time on the site, reduced ‘data analysis time’ and reduced cost fluctuations and data transfer costs	support operational efficiency, user empowerment, faster code trouble-shooting and more ‘accurate decision-making in complex service environments’
**Google Cloud** Welcome to Google Cloud Next ’24	faster processing of data sources faster, more accurate and optimized development through AI coding support ‘beneficial’ AI integration into workflows accelerates marketing and campaigning	support the most difficult workloads security protection of sensitive data dynamic and optimized resource management regulatory compliance management sustainability increased reliability and accuracy of Google Search faster evaluation ‘improve patient care’ ‘streamline supply-chains’ ‘increase’ and improve customer engagement ‘modernize shopping experiences’ enhanced cybersecurity employee productivity effective creative design support with teams regaining ‘time back to focus on high-level plans’. creative problem-solving
**Google** Agents	reducing time-intensive data transformations, model retraining or fine-tuning	unlocking a ‘myriad application and possibilities’ for agents to ‘not only understand the world but act upon it’ performance of a variety of tasks accuracy and reliability
**Google** AI Agents Companion	Agentic AI benefits compared with traditional systems: increased accuracy, faster task completion, better and dynamic coordination of multiple, complex tasks and errors, reduced hallucinations and bias, increased personalization and meaningful customer engagement, task-support in automation and knowledge management.	identify business trends streamline operational tasks speed up development cycles through more efficient identification of system bugs in-depth performance analysis ‘optimized content recommendations’, ‘deeper performance analysis’ and ‘fine-tuned campaigns’ for ‘better results’ (p. 38) in-depth and meaningful understanding of research information
**Google** AI for government	supporting the work of tax authorities employee productivity gains and saving time ‘attracting top-talent’ in the public sector (p.16) providing learning support	‘real’ GDP increases (p.17) average household income increases stimulate consumption reduce unemployment support employability ‘maximize return on investment’ (p.18) productivity gains in public administration and service delivery reduce bureaucracy and procedural delays fiscal deficit reductions projected use case benefits: accelerate and streamline infrastructure permits fraud monitoring and compliance streamline ‘committee decision-making’ (p.13) reduce ‘administrative burden’ of social workers (p.13) equitable access to healthcare diagnosis (p.14) accelerate drug discovery support ‘personalized learning pathways’ (p.15) accelerate content production, customer insights and customer reach ‘equitable’ and ‘reliable’ implementation of farmer programmes (p. 25)
		‘reduce delays’ and ‘enhance accessibility’ of justice (p. 26) optimizing the workflow and efficiency in airport operations accelerate growth of small businesses risk prevention of economic and physical casualties in disaster management (p. 53)
**DeepMind** AlphaFold: Five Years of Impact	broader access reducing discovery timelines lowering research costs	replicating ‘transformative potential’ of AlphaFold architecture and infrastructure in observed benefits to other domains (i.e. scientific discovery, Earth sciences, fusion).
**Microsoft** The Business Opportunity of AI	return on AI investment business value through faster AI deployment ‘customer satisfaction’ ‘employee productivity’ ‘market share’ increase ‘maximize organizational efficiency’	creating business value through faster AI deployment and shorter implementation rounds repeated business value through AI leadership’
**Microsoft** Transforming Public Sector Services through Generative AI	speed up time and increasing accuracy in managing citizen queries streamline employee workload and time through augmenting repetitive tasks	productivity gains economic benefits creative support evidence-based decision-making internal knowledge management
**OpenAI** Identifying and scaling AI use cases	minimize repetitive and manual tasks bridge knowledge-gaps saving time synthesize high amount of information faster trouble-shooting data analysis without coding skills high efficiency and product usability accelerate risk assessment	productivity gains increase employee satisfaction ‘optimize workflows’ (p. 8) ‘reduce skills bottlenecks’ and acquiring new skillsets (p. 7) ‘speed up innovation’ (p. 8) create ‘further interest in investment’ (p. 27)
**OpenAI** Introducing OpenAI for Government	saving time on repetitive work efficient time-allocation for ‘high-impact’ work improve the quality of services	employee satisfaction improving access to public services streamline data operations supporting national security interests (i.e. cybersecurity)
**Amazon Web Services** The generative AI advantage	reducing response times to queries customer satisfaction time-efficient and faster scaling of use cases reduce ‘development efforts’ (p.17)	competitive advantage in Gen AI applications greater efficiency greater productivity employee satisfaction creating new skills and roles
**Amazon Web Services** A Year of Progress	reduce administrative tasks efficient information access time-efficient project completion enhancing ‘analytical capabilities and operational efficiency’	‘accelerate innovation’ in education meeting ‘the academic community AI needs’ meeting different legal mandates on data requirements
**Nvidia** Demystifying Generative AI	not identified	speed up market entry with model customization hybrid-based infrastructure for ‘optimal’ ‘capex- and opex-based resourcing’ simplified management of resources with an AI centre of excellence
**Nvidia** AI-ready enterprise platform for the public sector	benefits of open-source and assurance creating accessibility to compute, tools and organization support	streamline data science process improved cost-effectiveness boosting AI development ‘modernize and accelerate infrastructure’ ‘serve the public’ ‘improve mission-critical outcomes’
**Nvidia** How Can GenAI Transform the Public Sector?	not identified	accelerate cost savings and efficiency ‘speed up ROI’ (p. 2)
**Anthropic** Building trusted AI in the enterprise	faster customer enquiries responding reduced coding time faster content creation work increased efficiency in back-office operations over 10% earnings from Generative AI accelerated product development reduced infrastructure costs time savings cutting down costs	model optimization to achieve ‘faster speeds’ in ‘high-quality results’ in AI assistance (p. 24) unlocking ‘new work’ and ‘frontiers’ for employees
**Anthropic** Economic Index report	‘fewer follow-up refinements’ and ‘growing confidence’ in task-delegation (p. 10) increase in gross domestic product per working-age capita Higher per capita Claude usage in education, administration, arts, life, physical and social sciences.	polarized labour outcomes with higher wages and risk of job displacement ‘fast-growth rate’ in knowledge-based tasks in education and science (p. 11) new product features enabling new work types shift to augmentation-focused usage with increase Claude usage per capita

## Data Availability

This article has no additional data.

## Footnotes

^1^
Our analysis is intended to apply to the entirety of AI as a technology, and thus the entire AI industry operating in the EU. However, we observe that the most pressing risks are posed by companies developing, deploying and providing key infrastructure for generative and frontier AI. Compared with traditional or predictive AI, these technologies use electricity, water, land and data at an unprecedented scale, and are predicted to cause substantial societal and economic disruption. They are likewise the subject of an intensifying international development race and thus benefit most from many of the subsidies described in §2. Development of the technology outside of a private industrial setting, for example, by public research institutes or universities, will also frequently fall outside of the scope of our analysis, where the latter does not receive subsidies. Uses of the term ‘innovation’ throughout the article should be understood in this private, commercial sense.

^2^
Throughout this article, we use the term ‘innovation’ to refer to research and development activities undertaken by technology companies. Our focus is on innovation understood in a commercial sense, not a broader scientific or academic sense.

^3^
Throughout this article, we discuss individual and societal interests on the one hand, and economic interests on the other. We interpret these categories through the lens of the EU tech policies reviewed in §4, which are underpinned by the EU Charter of Fundamental Rights among other foundational frameworks. Individuals enjoy a range of fundamental rights, some of which concern individual or social goods (e.g. right to equality and right to data protection), whereas others address economic goods (economic matters, e.g. right to property and freedom to conduct a business). This rough distinction between individual/social and economic interests (which also includes industrial interests) is reflected in the introductory text and recitals of the reviewed tech policies, with a balance being sought between them. It is in this sense that we use these categories. We do not examine or intend to make categorical claims about the overlap and causal relations between these types of interests, as doing so goes beyond the scope of the article. For readers interested in this area, we would refer them to the brief discussion in §3.3 and related works by economists such as Adam Smith and Karl Polanyi.

^4^
We define social well-being in relation to individual happiness as the contributions society receives from individuals to public goods. As discussed in §6, individuals with increased happiness are more likely to make positive contributions to society and public goods, and in doing so improve social well-being.

^5^
For context and background on recent political developments, see [12].

^6^
For examples of this type of mechanism in action, see [13].

^7^
For criticism of these simplification efforts, see [17, pp. 7–9].

^8^
For a US focus where a similar idea is currently discussed [28].

^9^
Specifically, the following will be exempted from mandatory environmental assessments: data centre projects, construction of EuroHPC supercomputer facilities, AI factories, giga factories, semiconductor projects, affordable housing development projects and projects on recharging points for electric vehicles can be excluded from mandatory environmental impact assessments, leaving it to Member States. See [15, Recital 14], [29].

^10^
Concerning the €1 billion savings for companies, see [29]. Concerning the total €180–325 billion cost, see [30, pp. 2 and 7]. For example, the EU has proposed to repeal a hazardous chemical database, which lists substances of concern in products on the EU single market.

^11^
Milan, for example, is already facing skyrocketing land prices. See [31].

^12^
According to a 2025 study, in Romania, a mere 21 jobs were promised, and yet only 10 were created. In North Holland, 2000 jobs were promised, but only a quarter materialized. See [33].

^13^
Sales tax exemptions are common for essential hardware, materials and energy to build data centres, including computers, wiring, air-conditioning units and electricity. New Jersey granted $77m in tax breaks and only one permanent job was created in return. See [35] and [36]. Illinois granted $38 million in tax exemptions for the creation of 20 jobs. See [37]. The article notes that ‘one Microsoft data center in Illinois received more than $38 million in data center sales tax exemptions but created just 20 permanent jobs…Of those, 37 have passed legislation specifically granting sales tax exemptions for data centers, and 16 of those states have granted nearly $6 billion in exemptions over the past five years’. Texas has also offered sales tax-based exemptions to data centres, see [38] and [39].

^14^
For example, an EU-wide target could be a standard ‘power usage effectiveness’ (PUE) score to be targeted by data centres across the EU. According to Andrew van der Haar of the Dutch Data Center Association, in reference to enforcement of the EED, ‘For the first three years, they are not going to give any penalties. Instead, they really want to work on the quality of the reporting, rather than penalizing companies for not reporting correctly’. See [33]. The current reporting framework does not provide an adequate level of transparency on general items in the reporting framework under the recast EED. See [44, p. 35]. For criticism of this approach to transparency, see [45, p. 30].

^15^
According to Koch, ‘Since 2014, FISA content requests to Meta have increased 2171%, while those to Google have risen 594%. Apple, though less transparent with its records, reported a 274% increase in such requests between 2018 and 2023’ [54].

^16^
A clear definition of what constitutes ‘digital sovereignty’ is not provided in these proposals. Civil society has long criticized this gap and warned that the lack of definitions for key concepts (e.g. sovereign data, (open/closed) models and/or infrastructure) can lead to proposals to improve digital sovereignty having the opposite effect by making the EU even more dependent on US technologies and infrastructure. See [56–58].

^17^
Civil society critics have suggested that the proposal is the latest attempt from industry to push for further deregulation rather than a genuine effort to realize digital sovereignty. See [61].

^18^
This would apply to multi-national groups with a turnover of at least €750 million; see [64].

^19^
These taxes can be significant. For example, in 2023, France collected €680 million, Italy €434 million, Spain €345 million and Austria €103 million. See [66].

^20^
Countries that introduced digital services taxes include France, Spain, Italy, Austria, Denmark, Hungary, Poland, Portugal and the UK. As an example, the UK taxes social media platforms, Internet search engines and online marketplaces. See [66].

^21^
For discussion of one example concerning a delayed fine for Google, see [68].

^22^
It should be noted that this report was published in September 2025 based on Code of Practice reporting by companies from 2024. Annual updates to the report are anticipated, so the compliance picture may change positively or negatively in the coming months and years.

^23^
It is worth noting that the Justice Department subsequently won its case against Google, but the case remains subject to appeal. See [94].

^24^
The ePrivacy Regulation was a proposed update to strengthen the 2002 ePrivacy Directive, which remains in force. See [103].

^25^
See Explanatory Memorandum Section 1 (Context of the Proposal), Art. 1, 3. The Digital Omnibus is a direct result of the Draghi and Letta reports in 2024, which were themselves heavily influenced by industry lobbying. See also [107–109].

^26^
At the time of writing, the Digital Omnibus remains in trilogue negotiations with a final vote anticipated by 2 August 2026 at the latest. References throughout this article refer to the original proposal published by the European Commission in November 2025.

^27^
[106, Art. 3–4], amending Arts. 4–5 of the GDPR and Arts. 3–4 and 15 EU Data Protection Regulation (EUDPR).

^28^
[106, Art. 3] amending Art. 88c GDPR.

^29^
[106, Art. 3] amending Art. 9 GDPR.

^30^
[106, Art. 3] amending Arts. 12–13 GDPR.

^31^
[106, Art. 3] amending Art. 4 GDPR.

^32^
[106, Art. 3] amending Art. 22 GDPR.

^33^
The Czech Republic, Italy and Germany were among the opponents.

^34^
For example, see [112], which states ‘[r]estoring Europe's competitiveness requires going much further than before in cutting red tape. Regulation must be proportionate, stable, coherent and technology neutral’.

^35^
Of course, deregulation should not be equated with an outright absence of governance. Where existing regulatory regimes are weakened, other forms of governance may fill the gap, including market incentives, corporate self-governance, professional self-governance and ethics and other mechanisms. However, the protections offered by such approaches may not be as robust or effective as the penalties and enforcement mechanisms available to regulators and public bodies [114].

^36^
Former UK Prime Minister Rishi Sunak now advises Microsoft and Anthropic. Former UK Chancellor George Osborne joined OpenAI in 2025 after leaving politics in 2017. Former No. 10 advisor Liam Booth-Smith joined Anthropic in 2025 after leaving UK politics in 2024. Former No. 10 advisor Cass Horowitz joined Google in 2025 after ending his stint in 2024. Nick Clegg, former UK Deputy Prime Minister and MP, moved from UK politics in 2017 to join Meta in 2018 and Hiro Capotla in 2025. For more, see [117].

^37^
Examples include automation of manual tasks, data analysis, or document processing; see [121, p. 4], [122, pp. 5, 41], [123, p. 4].

^38^
Amazon discusses existing public partnerships in North America and Australia here [130]; OpenAI has partnerships across the US government, including the Department of Defence, US Department of Energy National Labs, Air Force Research Laboratory, National Aeronautics and Space Administration (NASA), National Institutes of Health and the US Treasury; see [131].

^39^
A PricewaterhouseCoopers (PWC) report commissioned by Google claimed that productivity gains of up to 3% are possible; see also [131], [132, p. 12], [133].

^40^
For example, making information about benefits or government policies more accessible for citizens and residents, see [134–136].

^41^
For example, Microsoft predict that their products will speed up permitting processes for new homes and apartments. They also cite virtual assistants (e.g. chatbots) as useful for the public. See [138].

^42^
Examples include using AI to find new music or information, or send messages while driving. See [123, p. 4], [128, p. 4], [142, pp. 54–57].

^43^
PWC on behalf of Google, for example, states that GenAI will ‘reduce unemployment’ without explaining how. See [132].

^44^
Prainsack defines public value as ‘The public value of data use is high when it can be plausibly assumed that it will have clear benefits either for many people, for society as a whole or for future generations, and that no person or group is likely to experience significant and undue harm as a result of that use. Moreover, public value will regularly be more pronounced if the benefits are likely to materialize for underserved or marginalized groups, owing to the overall lower baseline and potential size of impact’.

^45^
Some well-publicized exceptions do exist to this general trend. See, for example, DeepMind's discussion of AlphaFold, which has advanced the ‘state of the art’ in protein folding; see [146].

^46^
In this regard, ‘public value’ is comparable to other ‘essentially contested concepts’, which allow for different, competing valid interpretations which can be rationally and genuinely held by different stakeholders. See [147].

^47^
For the full survey data, see [154].

^48^
It should be noted that some economists have suggested that many recent large lay-offs attributed by employers to AI automation may in fact have other causes, see for example [158–160]

^49^
For an overview of the Terms of Service of major GenAI companies and platforms, see [71,161]. In June 2026, Microsoft deleted this language from their Terms of Service, instead stating that ‘Copilot can make mistakes, and it may not work as intended. Do not use Copilot as a substitute for professional advice. Always verify the accuracy of information presented by Copilot before you rely on it. We are not responsible for any consequences that arise from your use of or reliance on Copilot’. For more, see [162–164].

^50^
For a legal discussion on AI companies not being liable for the outputs of their GenAI systems, see [71,161]. For a general overview, see [165].

^51^
For example, maximizing profit, increasing wealth, and the creation or disappearance of jobs and social inequalities are said to have causal relationships with economic growth. See [166, p. 72], [167, p. 147].

^52^
However, given that many AI companies are multi-national and headquartered in locations with favourable tax or regulatory conditions, the degree to which growth in the AI industry will lead to greater tax contributions in specific countries or regions remains an open question.

^53^
Adam Smith, considered by many to be the ‘father’ of capitalism, already recognized this tension in the 18th century. He understood happiness as ‘tranquillity and enjoyment’, and argued that ‘disorders of human life…arise from over-rating the difference between one permanent situation and another’, meaning that the pursuit of improvements to a society's standard of living can undermine its residents’ happiness. See [168, p. 149], [169, p. 310], [170, p. 71], [171–173].

^54^
Adam Smith, for example, observed that as soon as such key basic goods are guaranteed with sufficient wealth (including throughout old age), acquiring more wealth for its own sake should be avoided. Rather, he advises people to focus on the enjoyment of leisurely time and tranquillity [168,169, p. 310], [170, p. 9]. Other influential theorists acknowledge the tension between growth and happiness, and warn against the dangers of unfettered economic growth. John Stuart Mill, John Maynard Keynes and John Rawls agree that after a certain living standard is achieved, other pursuits should be prioritized over economic growth. Unfettered growth compromises happiness, leisurely time and justice. See [177, pp. 478–479].

^55^
Joseph Stiglitz, for example, has observed that rapid economic growth in the USA in recent decades has predominantly benefited the rich, with the ‘average’ American financially worse off and earning less in real terms than 60 years ago. See [178]. Policies on education, minimum wage, more stringent tax systems, workers’ rights and anti-discrimination laws are necessary to prevent inequality and increase the welfare of the public. See [167, p. 147]. Thomas Piketty has similarly examined how unfettered economic growth leads to wealth concentration; see [166, p. 25]. More recently, these phenomena have been referred to as the ‘K-shaped’ US economy, with a privileged few responsible for an increasing proportion of spending despite the ‘average’ person being worse off in terms of disposable income. See [179,180].

^56^
For example, through access to the technology, disruption to employment, regional impacts of data centres and other mechanisms. See [181–183], [184, pp. 3–8].

^57^
Adam Smith advocated for a ‘sufficientarian’ standard rather than an egalitarian one, observing that ‘no society can surely be flourishing and happy, of which the far greater part of the members are poor and miserable’. See [185, p. 78]. Modern economists have picked up and reinforced the importance of reducing inequality as an enabler of economic growth. See [169, pp. 315–317], [186, pp. 54–55], [187, pp. 39–41].

^58^
For example, to ensure essential goods, reduce inequality or guarantee ‘liberty’ and ‘security’ to enable ‘tranquillity’ and ‘flourishing’. See [169, pp. 309, 314].

^59^
The examples discussed below cannot offer a comprehensive picture of all AI-relevant policy. They do, however, show this balancing act in practice, and in some cases specify the concrete value technology can add to society.

^60^
For an example focused on the AI Act, see [190].

^61^
The AI Act predicts benefits for the following wide range of sectors: ‘healthcare, agriculture, food safety, education and training, media, sports, culture, infrastructure management, energy, transport and logistics, public services, security, justice, resource and energy efficiency, environmental monitoring, the conservation and restoration of biodiversity and ecosystems and climate change mitigation and adaptation’. See [195, Recital 4].

^62^
For discussion of environmental protections, see [203].

^63^
For a similar criticism, see Prainsack who proposes to use ‘data solidarity’ which ‘promotes the active facilitation of data uses that create public value without imposing significant risks’ to counter this problem [145, p. 112].

^64^
There are, of course, some notable exceptions, such as restrictions on automated individual decisions in the EU's 1995 Data Protection Directive. See [208].

^65^
For further discussion on the different levels of protection offered to data based on their degrees of identifiability and sensitivity, see [212].

^66^
The DA and DGA display similar risk mitigation strategies, albeit less directly. The DA seeks to reduce barriers to data sharing for the benefit of society, and thus is not solely about risk mitigation. See [196, Recital 2]. However, both the DA and DGA also address a range of risks posed by data sharing and governance arrangements.

^67^
For example, see [106, Recital 2].

^68^
The designation of some AI systems as ‘general-purpose’ in the EU AI Act would appear to reflect this fact. However, it cannot possibly fully regulate the risks posed by such a generalist technology. The Act is grounded in product safety and consumer protection regulation which, by definition, only covers a narrow range of risks. For discussion, see [200,201]. Further, it is worth remembering that not all potential risks of a technology can be foreseen. See [213, p. 1033], [214, p. 2].

^69^
For example, allocational harms arising from bias in the training data and outputs of predictive AI systems are both well-understood and relatively well-regulated by EU non-discrimination law, whereas for representational harms arising from bias in generative AI systems, scientific consensus and how best to apply relevant regulatory frameworks remain open questions. See [182].

^70^
The GDPR and AI Act (i.e. provisions on general-purpose models) are notable exceptions to this trend as horizontal legal frameworks.

^71^
See, for example, concerning regulation of acute and long-term speech harms [71].

^72^
Such initiatives include the ‘Centre for Humane Technology’ and ‘Time Well Spent’ movements (https://www.humanetech.com), United Nations ‘AI for Good’ initiative (https://aiforgood.itu.int/about-us/), AI4People project (https://ai4people.org/), ‘AI for Human Flourishing’ movement (https://hfh.fas.harvard.edu/ai-human-flourishing), ALLAI Academy (https://allai.nl/) and various ‘Human Centred AI’ initiatives (https://hai.stanford.edu/, https://hailab.ox.ac.uk/ and https://hcai.se/). Generally speaking, these initiatives move away from a sole focus on technological capabilities, and instead place human welfare as the central concern in technology development or governance.

^73^
It is worth noting that a related concept, that of ‘relationality’, has been applied to questions of data governance. For more, see [269].

^74^
With a focus on ChatGPT-4, see [338, pp. 1–3, 11].

^75^
For example, people may have fewer opportunities to experience the joy of learning, understanding and mastering a skill. The societal cost of having less capable doctors or other professionals in high-risk settings such as medicine, finance, law or economics must also be considered.

^76^
Here, ‘public value’ is proposed as a guiding principle to determine when data access is justified. Depending on public value of the data access, risk mitigation duties, prohibitions or even benefit sharing duties should be set with data donating communities. Similarly, biobanks have promoted the idea of creating public value in return for data access as well. See [248, pp. 113, 115], [249, p. 54].

^77^
Inspiration can be taken from movements like ‘slow technology’, see [362].

^78^
According to its creators, ‘The Happiness Curriculum is made up of compulsory 45-minute daily lessons with the aim of developing mindfulness, critical thinking, reflection, and social-emotional skills’. The curriculum also includes ‘Three units–Exploring Happiness through Learning and Awareness; Experiencing Happiness in Relationships through Feelings; and Happiness through active participation–based on a “triad of happiness” and the philosophy of coexistence are taught from nursery to grade 8’. For more information about how this is embedded in the classroom, see [364].

^79^
For example, how should impacts on individual happiness and social well-being be measured in practice? What is a reasonable level of such benefits to expect from individual companies in exchange for which types and degrees of subsidies? Should all AI products and services be subject to happiness requirements by default, or only those that have demonstrably benefited from subsidies? Should certain social benefits or antecedents of happiness be given greater weight than others, and under which conditions? Do regulators possess the necessary resources and capabilities to assess ‘happiness plans’ submitted by companies?

^80^
Indeed, a complementary policy response could be to adopt a shared public ownership model of AI companies through which all citizens are formally shareholders, similar to Norwegian partial state ownership of oil companies or Sen. Bernie Sanders’ recently proposed AI sovereign wealth fund. See [28].

## Appendix A. Reviewed AI industry documents

Tables 3 and 4 list AI industry documents (i.e. reports, sales materials, press releases, product briefings and other grey literature) we reviewed to determine the vision of general-purpose and generative AI currently being sold by industry. In some cases, the reviewed documents were published in partnership with other organizations, or on behalf of the company by third-party consultants or researchers. Research papers, technical reports, research blog posts and policy white papers were treated as out of scope except where explicitly used for sales or promotional purposes to ensure a narrow focus on promotional materials targeted at potential customers and policymakers. Documents were identified through manual searching of company websites. We reviewed documents published or sponsored by IBM, Google, DeepMind, Microsoft, OpenAI, Amazon Web Services, Nvidia and Anthropic. For each document, we describe its vision for AI (i.e. near- and long-term predictions about the value of generative AI), strategies for implementation and usage (e.g. advice, best practices and insights about generative AI to fulfil aims and objectives) and beneficial use cases.

With regard to vision, most of the reviewed documents describe a vision of generative AI focused on the return on investment and gaining competitive advantages. Described motivations include increasing financial returns, ensuring AI leadership and finding new ways to scale or speed up processes and improve efficiency. None of the reviewed reports focuses on longer-term predictions or visions for AI, with the exception of one document published by Google Cloud [122].

With regard to strategies, most focus on adoption of AI by a business or organization for optimizing or automating processes, or upskilling where success rate can be measured empirically (e.g. claims regarding process efficiency and faster deployment of new technologies). Comparisons are drawn between ML and generative AI ‘innovations’ focused on improved task efficiency, such as data analysis or document processing. Little attention is given to the reliability or social, ethical or legal factors of AI systems in sales materials, which may instead be dealt with predominantly in research, technical reports and policy white papers.^81^

With regard to use cases, the absence of descriptions of concrete, socially beneficial use cases in most of the reviewed promotional materials was notable. They lacked clear descriptions of mechanisms by which predicted social benefits will be delivered (i.e. more than ‘economic growth’, ‘competitiveness’ or ‘efficiency’ as being inherently good). One notable exception is a list maintained by Google Cloud which as of 22 April 2026 lists 1302 use cases of generative AI by organizations worldwide; however, even here concrete details of social benefits are lacking, with each use case receiving a one- to two-sentence description.^82^ The focus was instead on high-level descriptions of types of tasks or work that might support specific outcomes (e.g. automated document summarization to increases employee productivity). Across these higher-level descriptions, AI was said to improve customer satisfaction and interactions, employee productivity and efficiency of work processes.

## Appendix B. Measuring and mandating happiness in public policy

Although unfamiliar to many, ‘happiness’ and related concepts like ‘well-being’ already serve as guiding principles for public policy decision-making in many countries, balanced with economic growth or GDP. None of these examples can be categorically endorsed as the ‘correct’ approach to mandating happiness as a public policy goal owing to vagueness, controversies, implementation challenges, methodological criticisms and other flaws; for example, some approaches reductively equate happiness and well-being with economic growth. Nonetheless, their existence shows that the concept has some traction within policy-making internationally as a foil to a myopic focus on economic growth and GDP.

### B.1. Public policy mandates

Policy mandates to measure and improve well-being or happiness have been adopted in several countries. Perhaps the best-known case of a public policy happiness mandate comes from Bhutan. Happiness is explicitly measured through the Gross National Happiness (GNH) index, defined by nine happiness measures (or ‘bowls’) [367],^83^ which are further operationalized via 33 indicators [367].^84^ An individual is considered happy if they possess at least 66% in all the 33 weighted indicators and ‘bowls’ [369, p. 1].^85^ Bhutan conducts surveys every 3–5 years to assess these measures and indicators. Survey results help guide public policy decisions, the allocation of resources and aim to incentivize NGOs and businesses to focus more on happiness and well-being rather than purely economic growth [367].

Following Bhutan's shift from an absolute monarchy to a democratic constitutional monarchy, many policies and laws informed by these happiness measures have been introduced from 2008 onwards [368, pp. 492–493, 504]. For example, GNH measures are clearly reflected in Bhutan's current 5-year public policy plan, which prioritizes climate resilience, vocational training, education, healthcare, digital transformation, resilience and governance [370, pp. 4–7]. Their national AI strategy similarly shows influence from the GNH, focusing on ‘holistic wellbeing, sustainability and cultural preservation’ among other goals [371, p. 7]. This holistic approach (‘GNH centric design’) prioritizes AI in key areas like agriculture, tourism and energy, health, education and public services, natural resources and biodiversity, culture and language [371, p. 8].

Other countries have adopted similar mandates, but under the heading of ‘well-being’. A 2009 report by the Stiglitz–Sen–Fitoussi Commission on ‘the limits of GDP as an indicator of economic performance and social progress’ influenced several countries’ approaches to measuring and mandating improvements to well-being. The report advocates for the inclusion of universal ‘quality of life’ measures when assessing social progress [372].^86^

In 2017, Italy became the first OECD country to explicitly include considerations of well-being and happiness alongside economic considerations when taking public policy and budgetary decisions [374, p. 3]. Italy adapted the report's recommended universal measures into 12 indicators of well-being (table 2),^87^ which can be revised over time [374, p. 12].

In 2021, Ireland similarly developed a ‘Well-being Framework’ inspired by the Stiglitz–Sen–Fitoussi Commission, with 11 indicators measured (table 2) every 4–5 years [373], [376, p. 3], [377].^88^ Policymakers are directed to factor results into their decisions. Example affected policy measures include a carbon tax,^89^ revisions to the tax and welfare system and equality measures (e.g. around gender and age) [376, p. V].

Other approaches draw a simpler direct connection between well-being and economic considerations. For example, in 2019, New Zealand introduced a ‘Wellbeing Budget’ focusing on domestic challenges including mental health, domestic violence and child poverty, support for Māori and Pacific people (e.g. skills and income), digital transformation and productivity, and low-emissions business transformation [378, p. 6]. In 2025, the Public Finance Act 1989 was then amended to require ‘social, economic, environmental and cultural’ well-being considerations to be included in budgetary decision-making [379, para. 26 M]. The improvement of living standards (e.g. health, education, good working conditions, cultural protection, leisure time, friends and family, housing, environmental protection, firms and markets, and good governance) is also linked to ‘well-being’ and ought to guide policy and budgetary decisions [380]. Nepal has also mandated improved well-being in economic terms via its ‘Prosperous Nepal, Happy Nepali’ plan, which aims to make Nepal a high-income country by 2043. Nepal does not granularly measure happiness, but instead defines it broadly as the realization of higher living standards (e.g. higher income, better healthcare and education, physical infrastructure) and socialist values [381, pp. 4–5, 25].

It is worth remembering that, as discussed above (§5.3), economic considerations are relevant antecedents of happiness, for example, in relation to fulfilment of basic needs, but only to the point of diminishing returns. Further, they are not as strong predictors as other concepts like relatedness, competence and autonomy.

### B.2. Guidance and information for policymakers

In some countries, well-being and happiness are measured solely for informational and guidance purposes, rather than as part of a public policy mandate. OECD member states, for example, participate in the ‘OECD Well-being Framework’, which compares well-being between countries over time based on measurements across 11 dimensions (table 2) [382,383]. OECD countries do sometimes integrate well-being measurements into policy and budgetary processes, but not owing to a legal mandate [374, p. 14], [376, p. 3].

Similarly, the United Kingdom Office for National Statistics started collecting ‘well-being’ data in 2010 across 10 dimensions and 60 indicators (table 2).^90^ The well-being data are intended to inform policymakers and help make decisions, understand the impact of certain actions (e.g. building a new airport) [386], and otherwise guide the Treasury [384]. However, unlike Italy and Ireland, policymakers are not legally mandated to use the data to improve societal well-being; rather, it serves as guidance or a checklist to assess competing economic and well-being interests in a fair manner (e.g. the value of a measure and its cost) [387].

This section does not claim to offer a comprehensive picture or assessment of the efficacy of these public policy ‘happiness’ and ‘well-being’ strategies.^91^ However, we have shown that happiness as a public policy compass is not a completely foreign concept. We have surveyed a range of approaches to measure and improve the well-being of the public, ranging from policies that are inspired by religious values and connection with nature to relationships with people and opportunities for choiceful living. These must, of course, be evaluated alongside the concrete social problems facing regions, economic well-being indicators and the improvement of living standards and quality of life. The concepts of relatedness, competence and autonomy are self-evidently reflected, if not explicitly mentioned, in many of these general social policies.^92^
